# Filtering cells with high mitochondrial content depletes viable metabolically altered malignant cell populations in cancer single-cell studies

**DOI:** 10.1186/s13059-025-03559-w

**Published:** 2025-04-09

**Authors:** Josephine Yates, Agnieszka Kraft, Valentina Boeva

**Affiliations:** 1 Department of Computer Science, Institute for Machine Learning, ETH Zürich, Zurich, CH-8092 Switzerland; 2 ETH AI Center, Zurich, Switzerland; 3 Swiss Institute for Bioinformatics (SIB), Lausanne, Switzerland; 4 Department of Thoracic Surgery, University Hospital Zurich, Zurich, Switzerland; 5 Cochin Institute, Inserm U1016, CNRS UMR 8104, Paris Descartes University UMR-S1016, Paris, 75014 France

**Keywords:** MT-RNA, Data quality, Single-cell RNA-seq, Cancer, Drug resistance, Metabolism

## Abstract

**Background:**

Single-cell transcriptomics has transformed our understanding of cellular diversity, yet noise from technical artifacts and low-quality cells can obscure key biological signals. A common practice is filtering out cells with a high percentage of mitochondrial RNA counts (pctMT), typically indicative of cell death. However, commonly used filtering thresholds, primarily derived from studies on healthy tissues, may be overly stringent for malignant cells, which often naturally exhibit higher baseline mitochondrial gene expression.

**Results:**

We examine nine public single-cell RNA-seq datasets from various cancers, including 441,445 cells from 134 patients, and public spatial transcriptomics data, assessing the viability of malignant cells with high pctMT. Our analysis reveals that malignant cells exhibit significantly higher pctMT than nonmalignant cells, without a notable increase in dissociation-induced stress scores. Malignant cells with high pctMT show metabolic dysregulation, including increased xenobiotic metabolism, relevant to therapeutic response. Analysis of pctMT in cancer cell lines further reveals links to drug resistance. We also observe associations between pctMT and malignant cell transcriptional heterogeneity, as well as patient clinical features.

**Conclusions:**

This study provides insights into the functional characteristics of malignant cells with elevated pctMT, challenging current quality control practices in tumor single-cell RNA-seq analyses and offering potential improvements in data interpretation for future cancer studies.

**Supplementary Information:**

The online version contains supplementary material available at 10.1186/s13059-025-03559-w.

## Background

Single-cell transcriptomics studies have led to significant progress in our understanding of tumor biology, paving the way for the development of personalized medicine [1–4]. A crucial early step in processing single-cell RNA-sequencing (scRNA-seq) is implementing rigorous quality control measures to exclude observations that do not represent viable single cells. Following established guidelines [5–8], cells exhibiting a high percentage of mitochondrial RNA content (pctMT) are routinely excluded from the analysis. This practice is based on evidence linking high pctMT to dissociation-induced stress and necrosis [9–11]. However, recent studies have highlighted the limitations of these standard quality control (QC) filters, advocating for novel, data-driven QC metrics [12–15].

Moreover, pctMT has been closely linked to cell-specific metabolic activity, leading to substantial variability across different cell types and often surpassing the thresholds set by traditional filters [8, 14, 16, 17]. For instance, Montserrat-Ayuso and Esteve-Codina [12] argued that conventional mitochondrial filters may inadvertently eliminate healthy cells with high metabolic activity. Additionally, most studies linking pctMT with cell quality have been conducted on healthy rather than diseased tissue, whereas malignant tissues often exhibit higher percentages of mitochondrial counts due to generally elevated mitochondrial DNA (mtDNA) copy number [18] or the activation of the mTOR pathway [19, 20]. Hence, using a predefined threshold or median absolute deviations based on the entire cell population to filter out cells with high pctMT in cancer studies might inadvertently eliminate functionally and clinically important malignant cells.

Here, we set out to determine whether malignant cells with high pctMT in cancer indeed correspond to cells suffering from the dissociation-induced stress, empty, or broken droplets, or if they represent a viable and functional component of malignant cells that should be preserved for downstream analysis. By examining publicly available scRNA-seq cancer datasets, we demonstrate that elevated pctMT in malignant cells is largely independent of dissociation-induced stress and that including cells with high pctMT does not significantly compromise dataset quality. We further show that high pctMT malignant cells are metabolically dysregulated and associated with drug response and patient clinical features. Our findings complement current guidelines for processing scRNA-seq datasets and are likely to inform refined quality control strategies in future studies of human cancers.

## Results

### Malignant cells show a significantly higher percentage of mitochondrial RNA than healthy counterparts in samples across cancer types

**Fig. 1:**
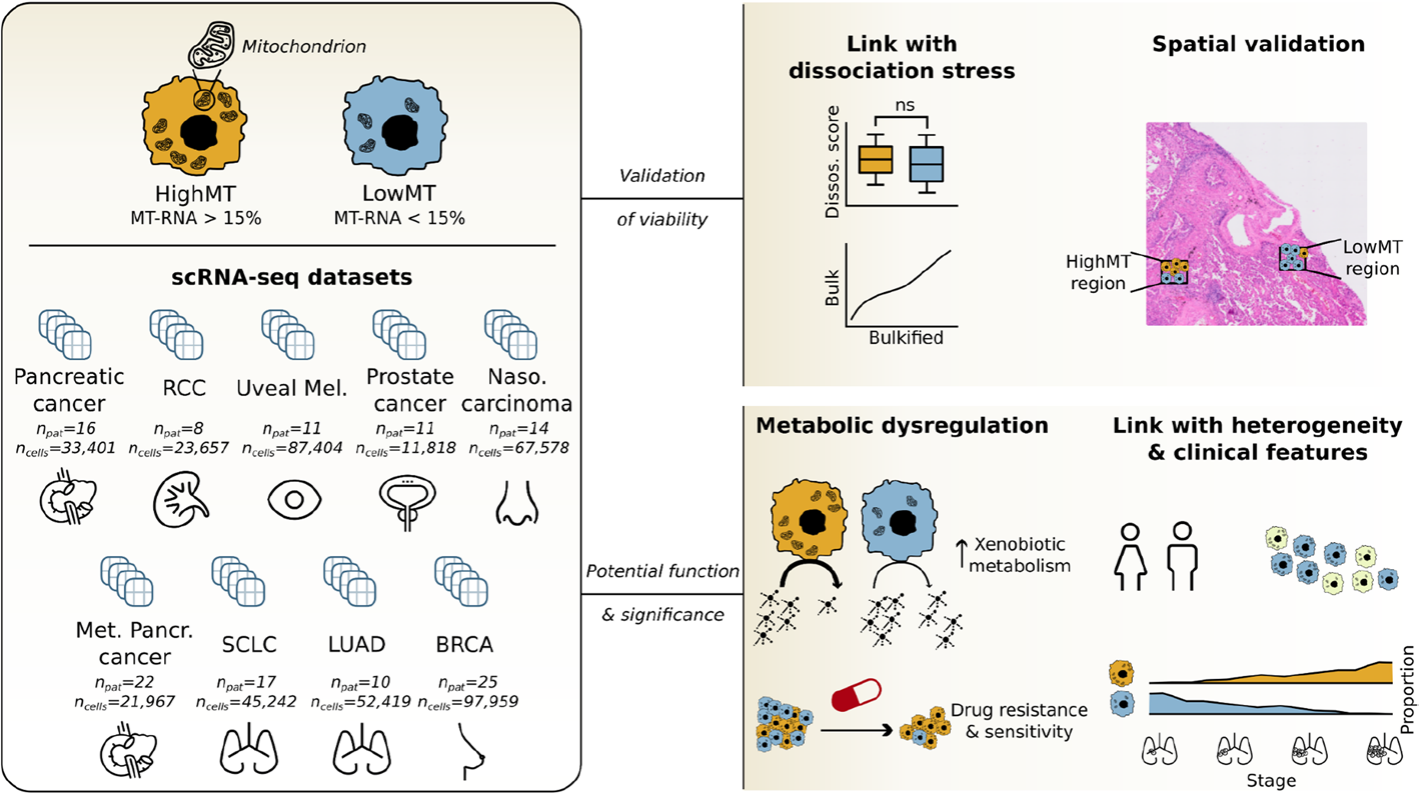
Study overview. We analyzed nine single-cell cancer datasets [4, 21–28] across 134 patients and 420,747 cells from various cancer types, categorizing cells by their percentage of mitochondrial-encoded gene RNA counts (pctMT), with cells above 15% designated as high mitochondrial content cells (HighMT). First, we examined potential links between pctMT and common artifacts, including dissociation-induced stress. We then confirmed regions of high-density malignant HighMT cells in Visium HD slides and explored metabolic dysregulation, notably an increase in xenobiotic metabolism in malignant HighMT cells. We linked cell line pctMT levels to differential drug resistance and sensitivity. Finally, we identified significant associations between pctMT and established cancer cell states, along with key clinical characteristics

To determine whether malignant cells exhibit a higher baseline pctMT, we analyzed pctMT levels in both tumor microenvironment (TME) and malignant cells across nine different studies: lung adenocarcinoma (LUAD), small cell lung (SCLC), renal cell (RCC), breast (BRCA), prostate, nasopharyngeal carcinoma (NPC), uveal melanoma, and primary and metastatic pancreatic cancers [4, 21–28], spanning the total of 441,445 cells across 134 patients, including 160,225 malignant cells (Fig. 1). PctMT levels were calculated based on the expression of mitochondrial genes detected in the dataset. These included at least the 13 protein-coding mitochondrial genes, with some datasets additionally incorporating mitochondrial transfer and ribosomal RNA genes (Additional File 1: Suppl. Table S1). We conducted extensive initial quality control (QC) without applying pctMT-based filtering. We evaluated whether this QC approach excluded potential low-quality cells by examining metrics typically associated with cell integrity, as outlined by Ilicic et al. [9]. Our analysis confirmed that the cells filtered out by our QC procedure consistently exhibited poor-quality metrics despite the QC not explicitly relying on pctMT (Additional File 2: Suppl. Fig. S1). Additionally, following recent studies recommending the use of *MALAT1* expression as a QC metric [12, 13], we compared the *MALAT1* expression between filtered and retained cells. We found that our filtering process effectively removed cells with high *MALAT1* expression, often associated with nuclear debris, and cells with null *MALAT1* expression, linked with cytosolic debris (Additional File 2: Suppl. Fig. S2).

**Fig. 2:**
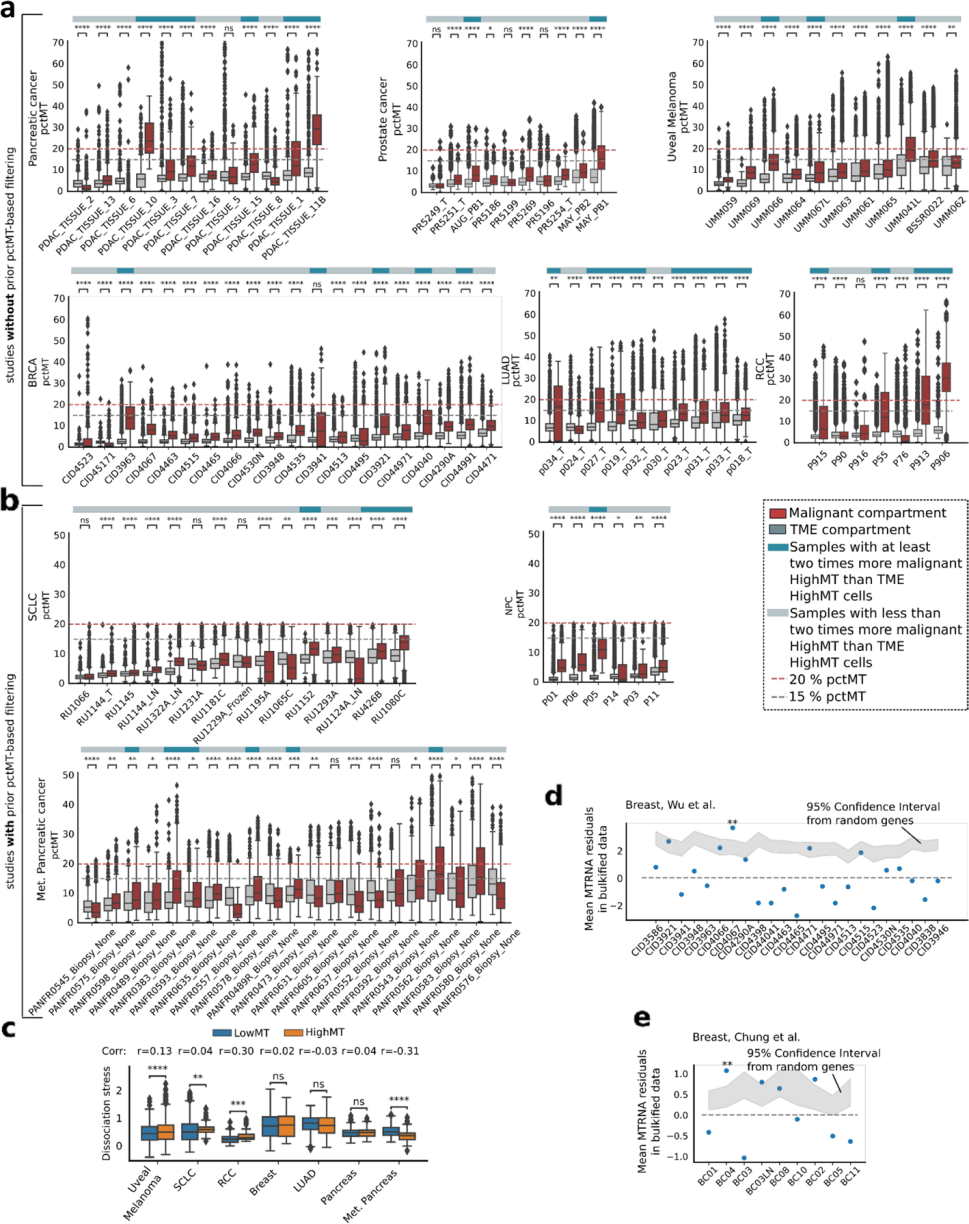
The malignant compartment of multiple cancer types contains cells with high mitochondrial-encoded RNA content.** a,b** Comparison of mitochondrial RNA percentage (pctMT) between tumor microenvironment (TME) and malignant cells across 112 patients in **a** unfiltered cohorts and** b** cohorts with prior pctMT filtering in original studies (Methods) [4, 21–26, 28]. Patients with too few TME or malignant cells are discarded for this analysis. Patients with more than double the proportion of HighMT malignant cells (pctMT > 15%) compared to TME and with over 15% of HighMT malignant cells are highlighted (blue bar above boxplots).** c** Distribution of the dissociation-induced stress scores estimated in HighMT and LowMT malignant metacells (pctMT < 15%) across the seven studies selected for the analysis (studies with at least two samples with at least 30% of HighMT malignant metacells). A dissociation stress meta-signature is defined using the common genes in three different dissociation stress signatures [10, 11, 34]. The point biserial correlation coefficient between the score and HighMT/LowMT status is indicated over the boxplots. **d,e** Mean of the residuals between the experimental and predicted expression of the 13 MT-encoded protein-coding genes for the paired bulk and single-cell data from the **d** Wu et al. [25] and **e** Chung et al. [35] cohorts. The relationship between bulk and bulkified gene expression is modeled by a polynomial regression. Residuals are computed as the difference between the ground-truth and the predicted bulkified expression. We use an empirical sampling scheme where we compare the mean residuals to that of randomly sampled genes (Methods). The 95% confidence interval of the mean residuals of randomly sampled genes is represented as the shaded gray area, and significance is reported based on Bonferroni-corrected *p*-values. RCC: renal cell carcinoma; SCLC: small cell lung cancer; NPC: nasopharyngeal carcinoma; LUAD: lung adenocarcinoma; BRCA: breast cancer; Met. Pancr. cancer: metastatic pancreatic cancer; TME: tumor microenvironment. Significance for a–c is computed with a Mann–Whitney U test. ns: $p>0.05$; *: $0.01<p\le 0.05$; **: $0.001<p\le 0.01$; ***: $0.0001<p\le 0.001$; ****: $p\le 0.0001$

We categorized cells as HighMT or LowMT based on their pctMT values, with those having pctMT above 15% designated as HighMT and those below 15% as LowMT. The value of 15% was chosen as the typical pctMT threshold range used in the non-cancer and cancer studies is 10–20% [24, 25, 29–33]. We detected significant variability in pctMT distribution between tumor microenvironment (TME) and malignant cells across patients, with generally higher median pctMT observed in the malignant cells in both filtered and unfiltered studies (Fig. 2a,b). Overall, 72% of samples (81 out of 112 patients used in this analysis, “ Methods”) had significantly higher pctMT in the malignant compartment (two-sided Mann–Whitney *U* test *p*-value < 0.05, Fig. 2a,b). Moreover, across studies of all cancer types, 10 to 50% of tumor samples exhibited a twice higher proportion of HighMT cells in the malignant compartment than in the TME (Methods), indicating a widespread presence of malignant cells that would typically be filtered out when the standard 15% cut-off on pctMT is used (Fig. 2a,b). The observed increase in pctMT in carcinomas could be partially explained by the natural variability in pctMT across cell types. Indeed, the basal pctMT of epithelial cells was generally higher than that of other TME components in most cancer types (Additional File 2: Suppl. Fig. S3-S11). However, in the majority of cases, the pctMT in the malignant compartment exceeded that of healthy epithelial cells (Additional File 2: Suppl. Fig. S3-S11).

### Malignant cells with high mitochondrial content do not strongly express markers of the dissociation-induced stress

We investigated the common hypothesis that the presence of malignant cells with high pctMT in scRNA-seq datasets is due to tissue dissociation protocol inducing cell stress. Utilizing dissociation-induced stress signatures derived from studies by O’Flanagan et al., Machado et al., and van den Brink et al. [10, 11, 34], we constructed a meta score based on genes found across all studies.

To determine whether the HighMT cells in the malignant compartment were associated with dissociation-induced stress without inflating the estimates of statistical significance, we computed metacell expression vectors for each study and excluded studies with only one patient with a twice higher proportion of HighMT cells in the malignant compartment [36]. The median number of cells per metacell ranged from 22 to 30 cells across the seven remaining studies (Additional File 2: Suppl. Fig. S12). In these seven studies, we compared the meta dissociation-induced stress scores between HighMT and LowMT metacells in both healthy and malignant compartments. The results revealed inconsistent patterns: one study indicated lower dissociation-induced stress in malignant HighMT cells, three showed no significant difference, and three showed higher dissociation stress in highMT cells (Fig. 2c). This variability persisted when scoring on a patient-specific basis (Additional File 2: Suppl. Fig. S3-S11). Notably, even in the studies where scores of the dissociation-induced stress were higher in the HighMT population of malignant cells, the effect size was small (maximum point biserial coefficient across studies < 0.3), suggesting dissociation-induced stress is unlikely to be the main driver of the HighMT cells in the malignant compartment.

To evaluate whether our QC procedure effectively removed cells stressed by tissue dissociation, or whether adding an additional pctMT filter would further reduce the presence of cells with high stress signature scores, we compared stress signature scores across three groups of malignant cells: cells filtered out by our in-house QC procedure, cells that would be excluded by a pctMT filter, and remaining cells (Additional File 2: Suppl. Fig. S13). Our analysis showed no significant increase in dissociation-induced stress scores among QC-passing HighMT cells, suggesting that the pctMT filter does not affect the proportion of cells with high stress signature scores. Therefore, applying a pctMT filter does not further reduce dissociation-related stress in retained cells.

To further demonstrate that dissociation-induced stress does not strongly drive elevated pctMT in the cancer cells passing other QC measures, we compared mitochondrial gene expression between paired bulk and scRNA-seq datasets from two breast cancer studies [25, 35]. Data from the bulk RNA-seq protocol, which does not require a tissue dissociation step, served as a control. We modeled the relationship between bulk and “bulkified” single-cell data and calculated the residuals reflecting the excess of gene expression from mitochondria in the scRNA-seq cells passing QC (Methods). In the Wu et al. cohort, only one out of 23 patients showed significantly higher residuals for mitochondrial-encoded genes than random nuclear-encoded genes (FDR-corrected p-value < 0.05); in the Chung et al. cohort, one out of nine patients showed significantly higher residuals (Fig. 2d,e). These results, consistent across models (Additional File 2: Suppl. Fig. S14), indicate that mitochondria-encoded genes are generally similarly expressed in bulk samples and QC-passing single-cell data, reinforcing the notion that HighMT malignant cells do not primarily arise from dissociation-induced stress.

### Spatial transcriptomics reveals subregions of breast and lung tissue with viable malignant cells expressing high levels of mitochondrial-encoded genes

**Fig. 3:**
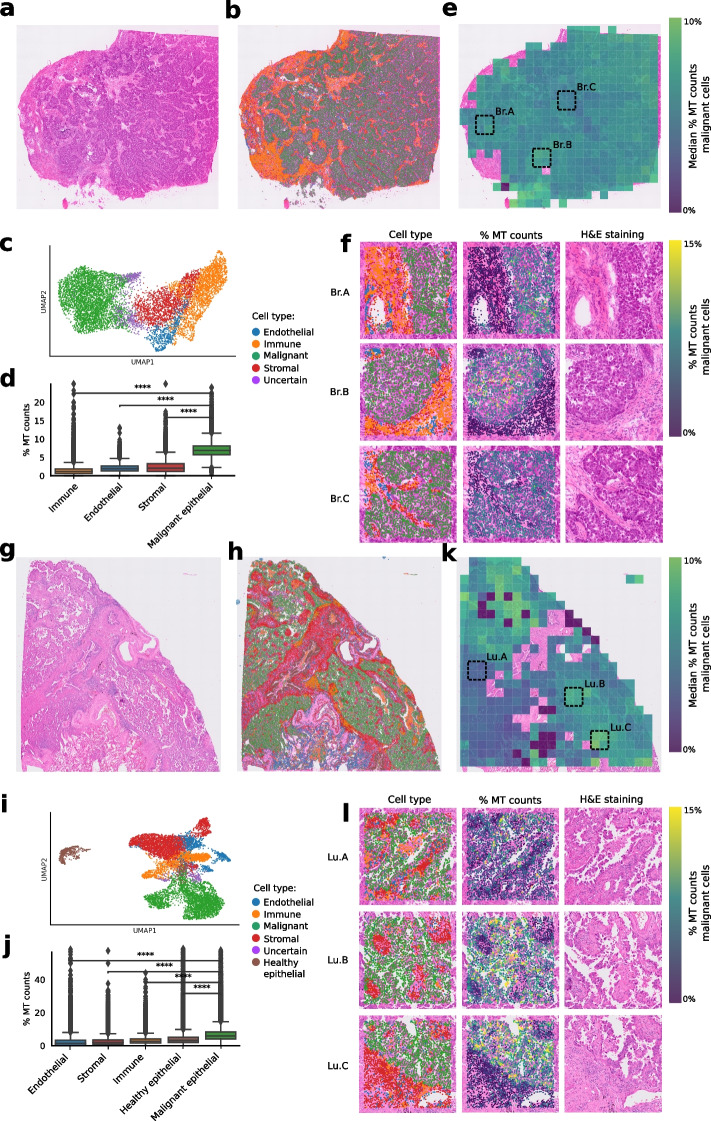
HighMT cells present varied distribution in spatial transcriptomics analyses of breast carcinoma and lung adenocarcinoma.** a** H&E staining of breast ductal carcinoma in situ (DCIS) analyzed with Visum HD. **b** H&E image overlay showing the annotated cell types. We use the log1p-normalized gene expression in cells segmented using bin2cell to perform Leiden clustering to define clusters, each aggregated into a “metacell” (Methods). Four primary cell type categories are identified, with copy number variation distinguishing malignant from healthy cells. **c** UMAP representation of the “metacells” in the tissue with cell type annotations. **d** Distribution of the pctMT (% MT counts) across cell types in bin2cell-estimated cells, analyzed by a Mann–Whitney *U* test (****: *p* < 0.0001). The plot is clipped at the 25% mark on the *y*-axis to better visualize the differences in distributions. **e** H&E image overlay showing median mitochondrial count percentages of malignant spots in the 1000 × 1000px patches. Regions with too few malignant cells are excluded. Breast regions of interest are marked as Br.A, Br.B, and Br.C. **f** Cell type annotations, pctMT values, and H&E staining of cells in regions of interest, with cell type annotations derived from metacell data. **g–l** Same analyses as **a–f** for lung adenocarcinoma (LUAD)

Despite the fact that we observed weak to no association between pctMT and dissociation-induced stress, we wanted to further exclude the hypothesis of the HighMT cells being necrotic. To address this, we examined Visium HD spatial transcriptomics data from one breast ductal carcinoma in situ (DCIS) patient (Fig. 3a–e) and one lung adenocarcinoma (LUAD) patient (Fig. 3f–j; “ Methods”).

Visium HD spots were transformed into segmented cells using the bin2cell tool [37], which leverages underlying H&E and immunofluorescence data for segmentation. These computationally estimated cells were aggregated into metacells, which were further used to annotate cell types using canonical marker expression and copy number variation analysis in DCIS (Fig. 3b,c) and LUAD (Fig. 3h,i). The uncovered copy number variation profiles reflected the published DCIS [38] and LUAD [39] profiles (Additional File 2: Suppl. Fig. S15). We computed pctMT using the 11 detected protein-coding MT genes (Additional File 1: Suppl. Table S1). Consistent with scRNA-seq findings, malignant cells exhibited a significantly higher pctMT than cells in the surrounding TME in both DCIS and LUAD, with numerous HighMT cells (pctMT > 15%) (Fig. 3d, j). Importantly, pctMT levels were not significantly correlated with the total detected counts in either malignant or healthy populations, ruling out a strong confounding effect of total detected counts on the analysis (Additional File 2: Suppl. Fig. S15).

To assess the spatial distribution of HighMT malignant cells, we calculated the median pctMT across 1000 × 1000px patches of malignant spots in both DCIS and LUAD tissues (Fig. 3e, k). This analysis revealed spatial variability, with localized regions showing higher median pctMT among malignant cells. In DCIS, we focused on three regions: Br.A and Br.B (high pctMT) and Br.C (low pctMT) (Fig. 3f). Each region showed consistent malignant cell morphology, but spot-level pctMT varied, with malignant cells displaying significantly elevated pctMT compared to adjacent non-malignant cells. Similarly, in LUAD, regions Lu.B and Lu.C showed markedly higher pctMT than region Lu.A (Fig. 3l).

The findings from spatial transcriptomics confirm that, independent of dissociation stress, malignant cells frequently display elevated pctMT and are variably distributed across tumor regions. This supports the conclusion that viable malignant cells with high pctMT constitute a prevalent component within tumors, observable even without dissociation-induced artifacts.

### Cells with high mitochondrial content express gene signatures associated with mitochondrial transfer and fission

To understand potential mechanisms driving higher pctMT observed in malignant cells, we explored the link between mitochondrial DNA and RNA content. Previous studies using single-cell and bulk tumor data have shown that transcription of MT-encoded genes positively correlated with the mitochondrial DNA content across healthy and diseased tissues [18, 40–43]. Moreover, Kim et al. analyzed matched mitochondrial DNA copy number and nuclear DNA data and observed that clones with increased MT-DNA to nuclear DNA ratio (MNR) were associated with higher transcription of mitochondrially encoded oxidative phosphorylation (OXPHOS) genes [40]. To assess whether a similar association is observed between MNR and pctMT in matching clones, we used available data from three ovarian cancer samples and six engineered hTERT cell lines from Kim et al. Overall, we observed a positive association between MNR and pctMT (Additional File 2: Suppl. Fig. S16).

**Fig. 4:**
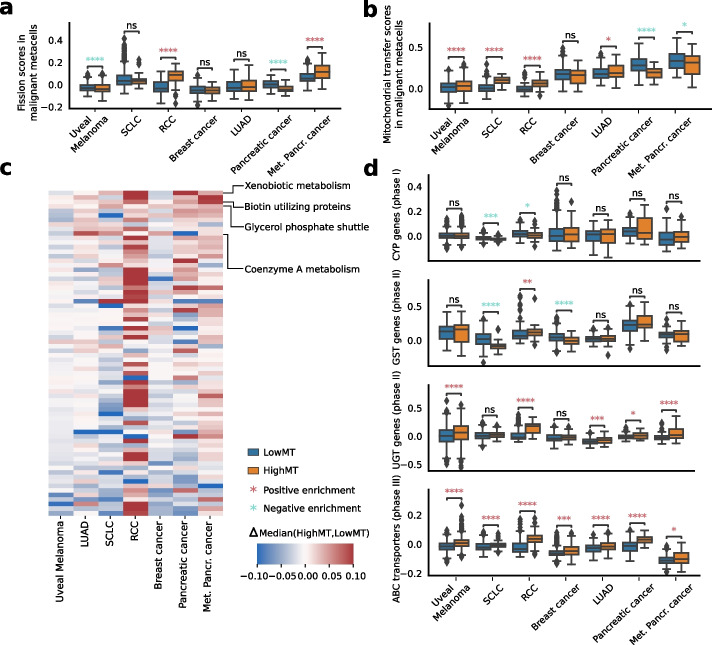
Transcriptomic and metabolic characterization of malignant cells with high mitochondrial content.** a** Distribution of metacell scores of mitochondrial fission across malignant compartments. **b** Distribution of metacell scores of mitochondrial transfer across malignant compartments. Significance is computed using a Kruskall-Wallis test. **c** Heatmap of the dysregulation of the 72 MitoCarta metabolic pathways. The hue represents the difference in median score of the pathway between the HighMT metacells and LowMT metacells. Pathways are ordered according to median difference. **d** Distribution of signature scores of genes involved in xenobiotic metabolism in the seven studies. The score of CYP genes (phase I), UGT and GST genes (phase II), and ABC transporters (phase III) are compared between HighMT and LowMT metacells for each study. Significance is computed using a Mann–Whitney *U* test. ns: $p>0.05$; *: $0.01<p\le 0.05$; **: $0.001<p\le 0.01$; ***: $0.0001<p\le 0.001$; ****: $p\le 0.0001$

Higher MT-DNA can result from several mechanisms, including mitochondrial fission [44] or horizontal mitochondrial transfer between TME and malignant cells [45–48]. We assessed the mitochondrial fission activity and the activity of mitochondrial transfer in malignant cells by scoring metacells with the gene ontology (GO) fission signature (GO:0090140), and a recently derived gene signature describing a cancer cell phenotype linked with receiving mitochondria from T-cells [49]. We observed significantly higher scores of one or both signatures in the HighMT malignant cells compared to LowMT ones in five out of seven studies (Fig. 4a,b), with the strongest effect observed in RCC for fission (point biserial correlation coefficient = 0.40, *p*-value < 0.001) and SCLC for mitochondria transfer (point biserial correlation coefficient = 0.36, *p*-value < 0.001). These results indicate that higher fission and/or mitochondria transfer from TME might be the driver of higher MT-DNA content and, as such, of higher MT-RNA expression in HighMT cells.

### Malignant cells with high mitochondrial content present dysregulation of metabolic pathways

Given the essential role of mitochondria in cell metabolism, we hypothesized that malignant cells with high mitochondrial content might exhibit metabolic dysregulation. To investigate this, we examined mitochondrial-related pathways curated by Mitocarta, which includes pathways involving nuclear-encoded proteins or RNAs that translocate to mitochondria [50] (Fig. 4c). We found that four consistently upregulated pathways in the studied cancer types were the glycerol phosphate shuttle (7/7 studies), biotin-utilizing proteins (7/7 studies), coenzyme A (CoA) metabolism (5/7 studies), and xenobiotic metabolism (5/7 studies), all with established roles in cancer [51–56]. Notably, oxidative phosphorylation — a core mitochondrial function — was significantly upregulated only in RCC and metastatic pancreatic cancer (Additional File 1: Suppl. Table S2), with other cancer types showing no shift or slight downregulation. These results indicate that HighMT cells display notable metabolic dysregulation.

### Cells with high mitochondrial content show increased xenobiotic metabolism through higher expression of drug-metabolizing enzymes and ABC transporters

Given our observation of the consistent increase of xenobiotic metabolism gene signature scores in malignant HighMT cells across cancer types and its implication in cancer therapeutic response [57, 58], we further characterized the activity of this pathway by evaluating the expression of genes involved in all three phases of xenobiotic metabolism: phase I cytochrome P450 (CYP) genes, phase II UDP-glycosyltransferase (UGT), and glutathione S-transferase (GST) genes, and phase III ABC transporters (Methods) [59].

We found that HighMT cells showed prominent upregulation of phase II and phase III genes (Fig. 4d). ABC transporters were notably significantly upregulated across all seven studies. UGT genes were also consistently elevated in all seven datasets, reaching statistical significance in five. In contrast, phase I genes showed no significant upregulation. This consistent pattern may reflect the known dependence of ABC transporter-mediated chemoresistance on mitochondrially produced ATP [60].

### Cell lines with higher mitochondrial content show resistance to metabolic drugs and sensitivity to targeting EGFR signaling

Given the high scores of xenobiotic metabolism gene signature in HighMT malignant cells, we further explored the link between the level of expression of mitochondrial RNA and the resistance of cells to commonly used drugs. We analyzed the association between the half-maximal inhibitory concentration (IC50) and mitochondrial content in cell lines from the Cancer Cell Line Encyclopedia (CCLE) [61]. Samples from CCLE showed diverse levels of expression of mitochondrial RNA, ranging from 4% median pctMT in glioblastoma to 14% median pctMT in head and neck squamous cell carcinoma (Additional File 2: Suppl. Fig. S17).

**Fig. 5:**
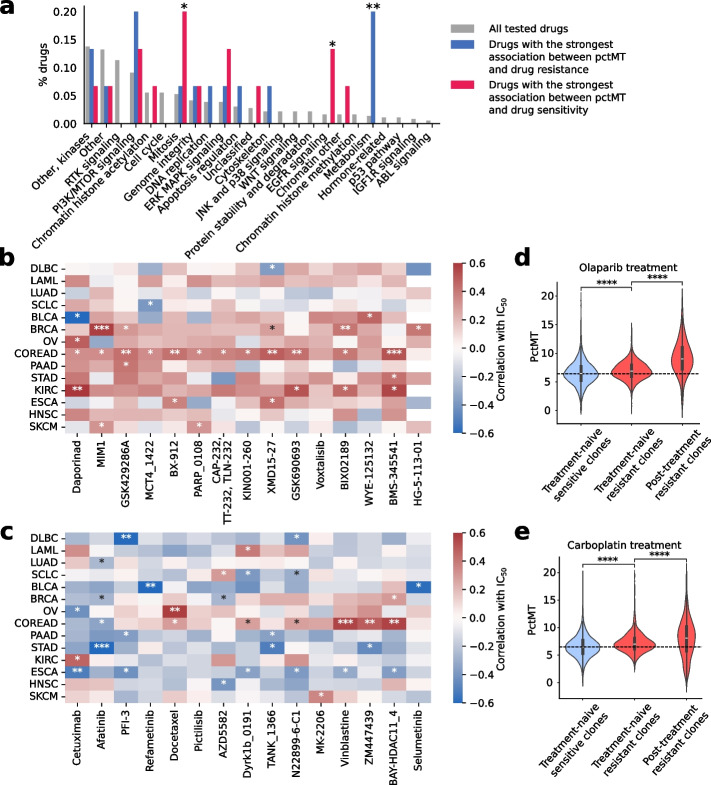
Cell lines with higher mitochondrial content show differential resistance and sensitivities to drugs.** a** Comparison of the function of the top 15 drugs with the highest association between pctMT and drug resistance (resp. drug sensitivity) and the set of tested drugs. All drugs tested on the CCLE are classified into categories according to their target. The fraction of drugs falling into each category is plotted. Significance is computed using a Fisher exact test. **b,c** Correlation between the pctMT of cell lines stratified by cancer type and IC50 of specific drugs for the top 15 drugs with the highest median correlation across cancer types (**b**) and the top 15 drugs with the lowest median correlation across cancer types (**c**). For each cancer type, Pearson’s correlation between pctMT and IC50 of all cell lines is computed. Significance is computed using Student’s *t* test. **d,e** Distribution of pctMT across the Kuramochi cell line’s treatment-sensitive and resistant clones, treated with Olaparib (**d**) and Carboplatin (**e**). Significance is computed using a Mann–Whitney *U* test. Dotted lines correspond to the median pctMT value in treatment-sensitive cells. *: $0.01\le p<0.05$; **: $0.001\le p<0.01$; ***: $p<0.001$. CCLE: Cancer Cell Line Encyclopedia

We observed a consistent and significant association between elevated pctMT and increased drug resistance, as indicated by higher IC50 values across cell lines with high pctMT (Methods). To confirm the robustness of these associations, we conducted an empirical permutation test, which demonstrated that the observed distribution of correlations significantly diverged from random, particularly in the tails (Additional File 2: Suppl. Fig. S17). The top 15 drugs with the highest median resistance across cancer types were significantly enriched in drugs targeting metabolism (Fig. 5a). These included Daporinad, which targets nicotinamide phosphoribosyltransferase (NAMPT) [62], BX-912, which targets PDK1 [63, 64], and CAP-232, which targets glycolysis. Many of the other drugs to which cells showed the highest resistance, although not directly associated with metabolism, targeted proteins involved in mitochondrial dynamics. This included MIM1, which targets MCL-1, involved in mitochondrial dynamics [65], MCT4_1422, which targets MCT4, a lactate transporter [66], XMD15-27, which targets CAMK2, linked to mitochondrial-dependent apoptosis [67], and BMS-345541, which targets IKK1, involved in mitochondrial network dynamics [68] (Fig. 5b).

We also found that higher pctMT in cell lines was consistently linked to higher sensitivity to drugs targeting EGFR signaling or mitosis (Fig. 4a). Specifically, higher pctMT correlated with increased sensitivity to common chemotherapy agents such as Docetaxel and Vinblastine (Fig. 5c). Interestingly, the highMT cells in most cancer types show an increase in expression of EGFR family genes, mostly *ERBB3*, which might partially explain increased sensitivity (Additional File 2: Suppl. Fig. S18). Although reports show that EGFR translocates to the mitochondria and is associated with metastasis in lung cancer [69–71], the exact mechanistic link between EGFR, increased mitochondrial content, and its role in carcinogenesis warrants further exploration.

To further validate the association between pctMT and drug response, we analyzed publicly available single-cell lineage tracing data from the high-grade serous ovarian carcinoma cell line Kuramochi, treated with carboplatin (DNA replication inhibitor) and olaparib (PARP1/2 inhibitor) [72]. Our CCLE analysis showed that pctMT was linked with resistance against DNA replication inhibitors, and with both sensitivity and resistance against genome integrity-targeting drugs (Fig. 5a). While carboplatin was not tested in CCLE, we observed a positive correlation between pctMT and olaparib IC50 in ovarian cancer cell lines (Pearson’s R = 0.35, *p*-value < 0.1), suggesting an association with drug resistance. Single-cell lineage tracing confirmed significantly higher pctMT in resistant clones compared to sensitive ones for both drugs, with pctMT further increasing in resistant clones post-treatment (Fig. 5d,e). We also analyzed lineage tracing data from the triple-negative breast cancer cell line MDAMB468, treated with afatinib [73], an EGFR inhibitor. Here, we found that pctMT in treatment-naive cells was significantly lower in the two most prevalent afatinib-tolerant clones (“dominant tolerant clones,” observed after afatinib treatment) compared to the sensitive ones (Mann–Whitney two-sided test *p*-value = 0.04, Additional File 2: Suppl. Fig. S19), agreeing with our results in CCLE data (Fig. 5c).

These findings support the association between pctMT and drug resistance, highlighting the importance of including cells with high pctMT in future analyses. However, to fully explore the mechanistic link between pctMT and treatment response, more systematic and extensive studies are required.

### Malignant cells with higher mitochondrial content are associated with previously reported transcriptional states and patient clinical features

Recent studies across various cancer types revealed the presence of diverse transcriptional profiles of malignant cells within individual tumors, and their association with patient treatment outcomes [74–76]. Hence, we investigated whether HighMT cells were associated with varied expression of previously reported transcriptional programs and states [77, 78].

**Fig. 6:**
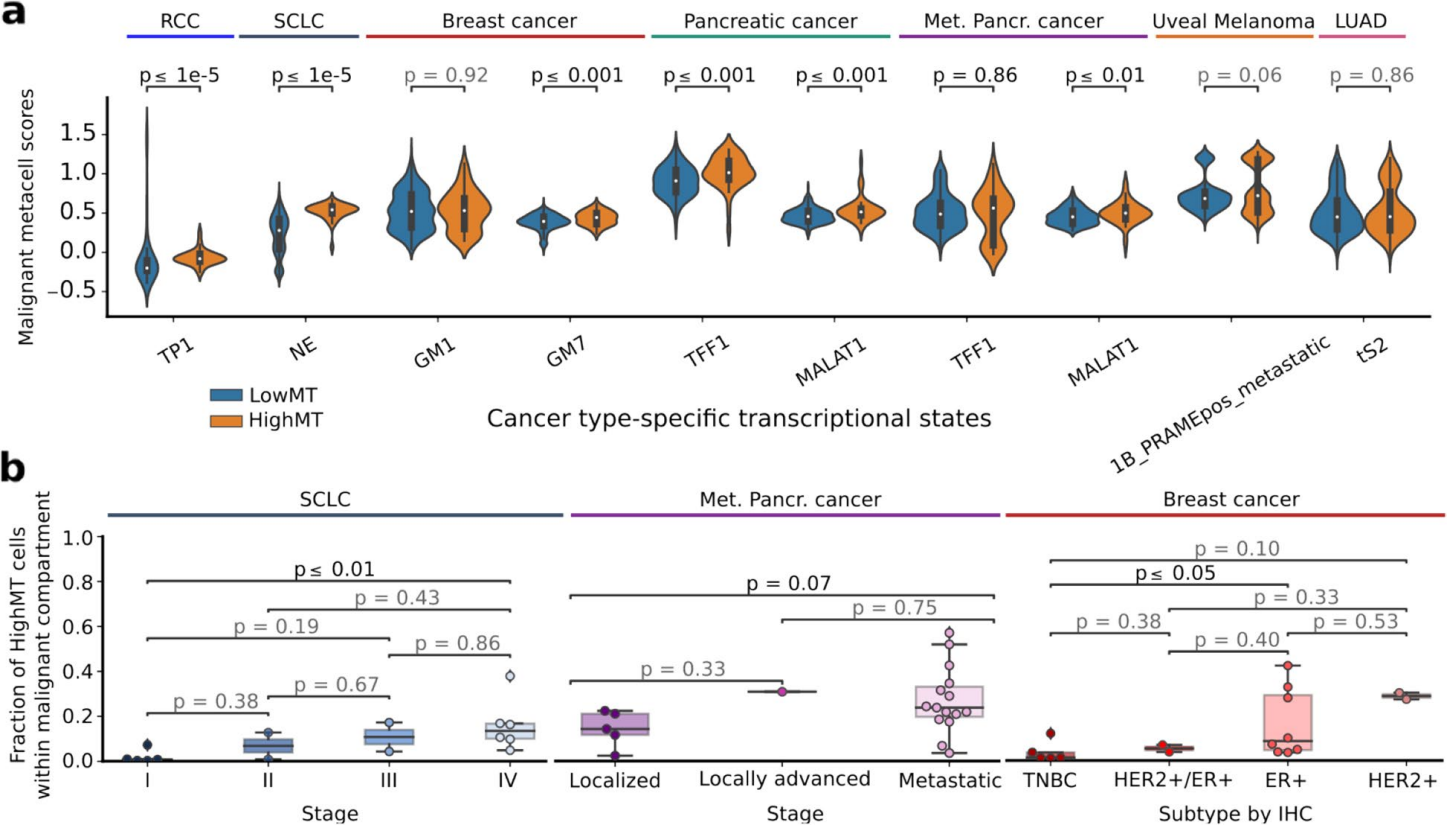
HighMT malignant cells are associated with transcriptional cell states and patient clinical features.** a** Distribution of scores of previously reported cancer type-specific transcriptional states across HighMT and LowMT cells. The cell states with the median cell-state scores higher in the HighMT than in LowMT cells are shown. Significance is computed using the Mann–Whitney *U* test on metacells. **b** Distribution of proportions of HighMT cells within malignant compartment per patient, across analyzed datasets and clinical features: stage in SCLC and metastatic pancreatic cancer, and IHC subtype in breast dataset. Significance is computed using the Mann–Whitney *U* test. TP1: tumor-program 1; NE: neuroendocrine-like program; GM1: estrogen response, hypoxia, tumor necrosis factor-α and p53 signaling and apoptosis program; GM7: hypoxia-related program; TFF1: mucin-related program; MALAT1: immune-rich program; 1B_PRAMEpos_metastatic: program expressed in class 1 *PRAME* positive metastatic cells; tS2: tumor state 2

We analyzed gene signature scores characterizing previously reported cancer type-specific transcriptional states in single-cell datasets of SCLC [79], breast [25], uveal melanoma [28], RCC [4], lung adenocarcinoma [80], and pancreatic cancer [81] single-cell studies. Malignant HighMT cells showed significant associations with scores of several reported transcriptional states (Fig. 6a, Additional File 2: Suppl. Fig. S20). Specifically, HighMT cells had significantly higher scores for tumor-program 1 (TP1) in RCC, neuroendocrine-like (NE) state in SCLC, mucin-related (TFF1 +) and immune-rich (MALAT1 +) states in primary and metastatic pancreatic cancer, and TNF-α and hypoxia-related state (GM7) in breast cancer.

Further, we investigated the link between the proportion of HighMT cells in the malignant compartment and patient clinical features in analyzed single-cell datasets (Fig. 6b). We observed a significant association between the proportion of HighMT malignant cells and stage in SCLC and metastatic pancreatic cancer, with a significantly higher proportion of HighMT malignant cells in more advanced stages (*p*-value < 0.1). In breast cancer, HighMT malignant cells were significantly enriched in the estrogen receptor-positive (ER +) subtype compared to triple-negative (TNBC) (*p*-value < 0.05). Taken together, these results show that retaining malignant HighMT cells in scRNA-seq analyses is crucial for accurately capturing tumor heterogeneity and relevant clinical correlations.

### Impact of filtering strategies on the retention of biological signals

Finally, to assess the impact of different filtering strategies on preserving biological signals, particularly those present in highMT cell populations, we applied three filtering approaches to the pancreatic cancer dataset from Steele et al. [23]. Specifically, we evaluated (1) traditional filtering with pctMT thresholds, (2) our proposed filtering approach that excludes pctMT thresholds, and (3) a data-driven quality control (DDQC) strategy described in [14].

For each approach, we analyzed the effects on downstream data, focusing on shifts in the distributions of xenobiotic metabolism and transcriptional state scores. Traditional pctMT thresholding introduced significant shifts in the expression of xenobiotic metabolism genes, which were highly expressed in HighMT cells identified in our study, compared to both our filtering strategy or DDQC filtering (Additional File 2: Suppl. Fig. S21). Additionally, traditional filtering led to a notably lower expression of the MALAT1 + transcriptional state, quantified by its score, relative to the other approaches.

These findings demonstrate that traditional filtering strategies can introduce artifacts, altering biologically meaningful signals in downstream analyses. Consequently, we recommend adopting our filtering strategy or modern DDQC approaches for single-cell cancer data analysis to ensure an accurate interpretation of biological phenomena.

## Discussion

Our findings provide evidence that malignant cells with high mitochondrial content, typically excluded from scRNA-seq analyses, constitute a metabolically dysregulated and functional subset. High percentages of mitochondrial-encoded gene counts have previously been linked to poor-quality cells, such as damaged droplets or cells affected by dissociation-induced stress [9, 10], leading many researchers to filter out cells exceeding a certain pctMT threshold using either static or dynamic criteria. However, recent advancements in data-driven quality control pipelines suggest that setting cell-type-specific data-driven QC thresholds can preserve biologically relevant cell populations, such as cardiomyocytes with high pctMT [14]. Our study corroborates these findings by showing that relaxing the pctMT filter reveals a group of cancer cells exhibiting dysregulated metabolic functions, notably upregulation of xenobiotic metabolism.

Of note, samples with equally high pctMT across all cell types, including healthy populations, should be carefully evaluated, as this pattern likely reflects technical artifacts or poor sample quality rather than true biological variation. In such cases, these samples should be excluded from the analysis to avoid misleading conclusions.

Importantly, our comparison of quality-filtered datasets, with and without pctMT thresholds, shows that including high-quality cells with high pctMT does not affect the overall distribution of dissociation-induced stress scores.

Consistent with previous literature [40], we found that HighMT populations could at least partially be explained by higher MT-DNA content. Higher MT-DNA might be caused by increased mitochondrial fission or horizontal mitochondrial transfer, as previously described [49], and linked to high pctMT in our analysis across several datasets. Hence, the presence of HighMT populations, rather than being caused by poor-quality cell capture in the single-cell protocol, can be due to a biologically driven increase in MT-DNA content.

We observed general metabolic dysregulation and upregulated activity of several processes, including xenobiotic metabolism, in HighMT malignant cells. This was mirrored by increased resistance to metabolic drugs in cell lines with high pctMT, suggesting clinical relevance in patient stratification and potential new avenues for combined therapies. These results, together with the association between HighMT cells and previously described transcriptional states, the overrepresentation of HighMT cells in patients of specific molecular subtypes, and the correlation between the proportion of HighMT cells and tumor stage, suggest the significant role of high pctMT malignant populations in cancer and the importance of including them in analyses.

When further investigating the potential function of malignant HighMT cells, we found that these cells exhibited upregulation of phase II and III genes involved in xenobiotic metabolism, with ABC transporters consistently upregulated across multiple studies. The interdependence between ABC transporter-mediated chemoresistance and mitochondrial ATP production, as highlighted in recent studies [60], may explain this consistent association. Given the limited effectiveness of ABC transporter inhibitors in reversing drug resistance in clinical settings [55], combining these inhibitors with mitochondrial inhibitors could be essential for overcoming resistance. Moreover, our findings using CCLE and lineage-tracing data reinforce the association between pctMT content and drug response, highlighting the importance of including malignant cells with high pctMT in analyses, as they could be clinically relevant for optimizing treatment strategies and improving patient stratification.

Several limitations should be acknowledged. First, since we used publicly available data, it was difficult to collect comprehensive datasets with no filters applied on pctMT during data preprocessing. Consequently, some of our datasets do not contain cells with very high pctMT, as these cells were prefiltered. This limitation makes it difficult to capture the full range of HighMT malignant cells, as some functionally or clinically relevant cells could have been excluded, potentially restricting the biological interpretation of HighMT malignant cells. However, we identified recurrent features of HighMT malignant cells across both unfiltered and filtered datasets, suggesting that the observed properties are a consistent and widespread aspect of these cells. Second, while we utilized previously identified dissociation-induced stress signatures to estimate the stress levels in cells with high pctMT, we lacked definitive ground truths (e.g., FACS-sorted stressed cells). Thus, our conclusions regarding the dissociation stress-pctMT relationship require further experimental validation. Third, our spatial analysis of co-existing HighMT and LowMT regions was limited to two samples, restricting the generalizability of our findings. Fourth, the number of probes used to detect mitochondrial-encoded genes in the Visium HD platform was one per gene, lower than the median of three probes per gene in the probe set, potentially affecting the detection of mitochondrial gene expression. Fifth, the signature we used for horizontal mitochondrial transfer was limited to transfer from T-cells and thus did not take into consideration potential horizontal transfer from other TME compartments. Finally, the link between pctMT and drug resistance and sensitivity was mostly conducted on cell lines, warranting further validation. While we also incorporated lineage-tracing data from two cell lines, to fully dissect the mechanistic link, extensive additional experimental and computational analyses are necessary.

## Conclusions

This study is the first to establish that in cancer scRNA-seq datasets, malignant cells with high pctMT, usually filtered out by standard QC procedures, are not solely associated with dissociation-induced stress or poor-quality droplets, but represent distinct, functional malignant cell subsets with altered metabolic functions and potentially differential drug responses. The inclusion of HighMT cells in cancer studies is crucial for improving the accuracy of patient stratification and identifying novel therapeutic targets. Moving forward, we recommend adopting more lenient or data-driven pctMT thresholds for scRNA-seq [14, 15] or spatial transcriptomics [82] to prevent the loss of valuable biological insights that may contribute to advancements in cancer research and treatment.

## Methods

### scRNA-seq preprocessing

We performed stringent quality control on a patient level across the nine included studies: uveal melanoma [28], small cell lung cancer (SCLC) [24], lung adenocarcinoma (LUAD) [27], renal clear cell cancer (RCC) [4], breast cancer (BRCA) [25], prostate cancer [21], nasopharyngeal carcinoma [26], pancreatic [23], and metastatic pancreatic cancer [22]. We followed the standard processing guidelines described at https://www.sc-best-practices.org/preprocessing_visualization/quality_control.html, excluding steps that involved using the percentage of mitochondrial counts as a quality measure. Notably, some studies had already filtered out cells with less than 20% [21, 24–26] or 25% mitochondrial counts [4]. Notably, the lung adenocarcinoma, renal cell carcinoma, and prostate cancer datasets contained cell type annotations only for cells kept in their study but provided raw counts for unfiltered cells; we thus assigned cell types using Leiden overclustering and majority voting of the cell types present in the cluster. For the breast cancer dataset, we retrieved raw FASTQ files from the European Genome Archive (EGAD00001007495), and ran gene expression quantification using CellRanger (v.9) to obtain raw counts for unfiltered cells. Cell type annotations kept in the original study were used to assign cell types, as described above. In all studies, if cells could not be assigned cell types using this procedure, they were removed from the analysis.

First, we removed all cells per patient that were more than 5 median absolute deviations from the median of either the log1p total number of counts in the cell, log1p genes expressed in the cell, or the percentage of counts falling in the top 50 genes. We also excluded cells with fewer than 1500 total counts, more than 50,000 total counts, and fewer than 500 genes expressed. Then, we identified and removed putative doublets using Scrublet [83].

Next, using the annotated cell types, we inferred copy number variation (CNV) with inferCNV (https://github.com/icbi-lab/infercnvpy). We clustered the cells in CNV space using the Leiden algorithm, assigning clusters a malignant CNV status if more than half of the cells mapping to the cluster were originally annotated as malignant; otherwise, we assigned a non-malignant CNV status. We removed cells with discordant CNV and transcriptomic identity from downstream analyses. For further analysis, we used the counts per 10 k transcripts (CP10K) transformation followed by log(1 + x) (log1p) transformation.

To compare different filtering strategies, we implemented the following approaches:

- Threshold-Based Filtering: This approach follows standard quality control (QC) procedures in Scanpy. Cells with fewer than 100 expressed genes, genes expressed in fewer than 3 cells, cells with > 15% mitochondrial gene content, and predicted doublets identified using Scrublet.
- Data-driven quality control (DDQC): We applied the method proposed by [14], following the provided tutorial and using default parameters for filtering.

### Annotating patients with more than double the proportion of HighMT malignant cells compared to TME HighMT cells

For each study, we compared the distribution of the percentage of transcripts mapping to mitochondrial-encoded gene (pctMT) between cells from the tumor microenvironment (TME) and the malignant cell compartment. We assigned cells to a high mitochondrial content status (HighMT) if they presented > 15% pctMT; otherwise, we considered them low mitochondrial content (LowMT). We compared the odds ratio of HighMT cells in the malignant and TME compartments in the rest of the samples using the formula:

$$OR = \frac{\frac{n(HighMT,mal)}{n(LowMT,mal)}}{\frac{n(HighMT,TME)}{n(LowMT,TME)}}$$

We classified patients as cases if they (*i*) had an OR > 2 and (*ii*) had at least 15% of HighMT cells in the malignant cell compartment; other patients were assigned to controls. For the patient-specific analysis, we removed patients that contained less than 30 malignant or TME cells, and patients that had less than 20 HighMT cells, thus resulting in 111/151 patients. We included only studies comprising more than one case for further analysis.

### Quality metrics and dissociation-induced score computation

The study by Ilicic et al. [9] identified seven metrics capable of discriminating between good quality cells and empty/broken cells in a cell-type and technology-agnostic manner, including the Gene Ontology terms *Cytoplasm* (GO:0005737) and *Mitochondrially localized proteins* (GO:0005739), and *mtDNA encoded genes* (equivalent to pctMT) and *Transcriptome variance*. To assess the expression of a gene signature representing GO terms, we applied standard Scanpy scoring [84]. To evaluate transcriptome variance, we calculated the variance per cell using log1p-CP10K-transformed data. We compared these scores between cells filtered out using our quality control (QC) procedure and those retained for downstream analysis.

To construct a dissociation-induced stress score, we aggregated signatures from three external studies. O’Flanagan et al. [10] derived a dissociation stress signature from patient-derived breast cancer xenografts, cell lines, and patient cancer cells using 37-degree collagenase dissociation. Machado et al. [34] developed a dissociation stress signature based on liver and muscle tissue samples, while Van den Brink et al. [11] derived a dissociation stress signature using muscle stem cells. To create a meta-dissociation stress signature, we compiled genes that were consistently found across all three dissociation stress signatures. Cells in our dataset were scored for this meta-dissociation stress signature using standard Scanpy scoring methods.

### Metacell computation

We aggregated single cells of the same type from all 151 patients sequenced through scRNA-seq into metacells to reduce sampling noise and capture underlying transcriptomic distributions, as introduced by Baran et al. [36]. Indeed, using metacells instead of single cells helps mitigate statistical inflation in single-cell RNA-seq data by aggregating highly similar cells into robust groups, thereby reducing noise from technical variability and sparsity in lowly expressed genes. This approach preserves the biological heterogeneity of the dataset while providing more reliable and stable measurements for downstream analyses. For all remaining seven studies, we implemented metacell aggregation using the Python metacells package (https://github.com/tanaylab/metacells). Metacells were defined as disjoint and homogenous groups of transcriptomic profiles that could potentially arise from the same underlying distribution.

Metacells containing more than 30% of cells with high mitochondrial content were categorized as HighMT metacells, while metacells containing more than 50% malignant cells were classified as malignant. These metacells underwent similar processing as the original scRNA-seq data, including scoring for dissociation stress using the meta-dissociation stress signature applied to log1p-CP10K transformed data. This approach allowed us to analyze and compare transcriptomic profiles at a more aggregated level, focusing on groups that potentially share similar biological characteristics.

### Bulk versus bulkified analysis

DNA library preparation for bulk RNA-seq does not include a tissue dissociation step. Therefore, we compared the expression of mitochondrially encoded (MT-encoded) genes between paired bulk RNA-seq and single-cell RNA-seq datasets to assess the potential effects of dissociation-induced stress on MT-encoded gene expression. Specifically, we utilized two datasets with paired single-cell and bulk data: the breast cancer datasets from Wu et al. [25], sequenced using 10X technology, and Chung et al. [35], sequenced using Smart-seq2.

The Wu et al. dataset underwent processing using our standard pipeline, while the Chung et al. dataset, due to its low cell count per patient, was analyzed collectively rather than on a per-patient basis. We used the Fragments Per Kilobase of transcript per Million mapped reads (FPKM) measure of gene-length corrected gene expression for bulk data from Wu et al. dataset; for Chung et al. we used the provided transcript per million (TPM) estimates.

We performed bulkification, i.e., aggregating single-cell measurements into one vector of gene expression per patient to mimic bulk data. Given Smart-seq2 is not naturally gene-length corrected as 10X measurements are, we used the TPM transformation for Smart-seq2 data while we used raw counts for 10X. For Wu et al., we summed raw counts across cells per patient followed by log1p normalization, while for Chung et al., we computed the mean TPM expression across cells per patient.

Due to inherent differences in noise and dropout rates between single-cell and bulk data, direct comparison of bulk and bulkified data is challenging. To model their relationship, we employed polynomial regression, varying degrees from 1 to 6 and evaluating the coefficient of determination (R2) for each. We selected the optimal model complexity based on the elbow of the R2 curve, where further increases in degree yielded minimal R2 improvement.

To assess similarity in MT-encoded gene expression between bulk and bulkified data, we trained a model excluding MT-encoded genes and computed residuals of predicted *vs.* observed bulkified expression for MT-encoded genes. Given their consistent high expression, MT-encoded genes often resulted in higher residuals, potentially affecting model fit. To statistically evaluate these residuals, we performed an empirical test. We randomly sampled genes from the top 500 most expressed genes in each dataset 500 times, trained models on the remaining genes, and computed residuals for these random genes. We calculated one-sided *p*-values based on how frequently residuals for these random genes exceeded those for MT-encoded genes, setting significance at 0.05.

This methodology allowed us to robustly compare MT-encoded gene expression profiles between bulk and bulkified data, providing insights into potential impacts of dissociation stress on transcriptomic measurements in single-cell RNA-seq studies.

### Spatial transcriptomics Visium HD processing and analysis

For data acquisition, we downloaded two Visium HD samples from the 10X Genomics website: a fresh frozen sample from a patient with breast ductal carcinoma in situ (DCIS) and a formalin-fixed paraffin-embedded (FFPE) sample from a lung adenocarcinoma (LUAD) patient.

To approximate single-cell expression, we utilized the bin2cell tool [37], following its tutorial (https://nbviewer.org/github/Teichlab/bin2cell/blob/main/notebooks/demo.ipynb). The data were first destriped, and segmentation was performed using both H&E and immunofluorescence data with Stardist, applying recommended parameters to estimate cell boundaries. Counts were normalized using counts per 10 k (CP10k) normalization, followed by log1p normalization.

Given the sparse and highly correlated nature of Visium HD measurements at the single-cell level, we conducted the analysis in terms of “metacells,” or clusters of spatially redundant spots representing aggregated cellular measurements. To construct metacells, we applied Leiden clustering to the 15-nearest neighbor graph, leading to 8884 and 8682 metacells in DCIS and LUAD, respectively. These metacells underwent the same CP10k log1p normalization and Leiden clustering as individual spots.

We used canonical marker scoring via Scanpy to assign cell types to each metacell. For LUAD, marker genes were based on major lung compartments from a recent lung cell atlas [29]: epithelial markers (*FXYD3*,* EPCAM*,* ELF3*), endothelial (*CLDN5*,* ECSCR*,* CLEC14A*), immune (*CD53*,* PTPRC*,* CORO1A*), stromal (*COL1A2*,* DCN*,* MFAP4*), and neuroendocrine (*CELF3*,* SLC6A17*,* CDK5R2*). For DCIS, we used markers from a recent single-cell study [85] identifying epithelial (*EPCAM*,* KRT7*,* KRT8*), immune (*CD3D, CD3E, CD79A, CD79B, CD19, MS4A1, CD3G, JCHAIN, MZB1, LYZ, CD68, FCGR3A*), endothelial (*PECAM1, VWF, CLDN5, CDH5, FLT1, RAMP2*), and stromal (*COL1A1, DCN, COL1A2, C1R, ACTA2*) compartments. Cell type assignment within clusters was based on maximum average scoring.

To profile copy number variation (CNV), we applied inferCNV (https://infercnvpy.readthedocs.io/en/latest/index.html), using presumed non-malignant metacells as the reference. Metacells were clustered by CNV profile, and each cluster was categorized as malignant or healthy based on average CNV scores. Final annotations were refined such that healthy CNV epithelial cells were labeled as “healthy” in LUAD and “uncertain” in DCIS, while TME cells with malignant CNV profiles were marked as “uncertain.”

Cell types were assigned based on the corresponding metacell annotation. We compared pctMT medians between malignant and TME cell types using a Mann–Whitney *U* test. The spatial distribution of pctMT in malignant cells was assessed by computing median pctMT in 1000 × 1000px regions; regions containing fewer than 10 malignant cells were excluded from further analysis.

### Association of pctMT with mitochondrial DNA content

To investigate whether the pctMT was linked to the mitochondrial DNA (mtDNA) content in single-cell data, we used matched single-cell RNA and WES data from Kim et al. [40]. The mtDNA content was evaluated using mtDNA to nuclear DNA ratio (MNR), i.e., the number of mtDNA copies per average haploid nuclear genome. Using the clone annotations called by authors, we compared the distribution of pctMT in clones with their distribution of MNR.

### Mitochondrial transfer and fission

We investigated the hypothesis that higher mitochondrial content in cancer cells may be attributed to horizontal mitochondrial transfer from cells within the tumor microenvironment (TME), as suggested by several studies [49, 86, 87]. To quantify the extent of mitochondrial transfer, we employed a signature derived from Zhang et al. [49], which characterizes mitochondrial transfer events. Similarly, to evaluate mitochondrial fission, we used the Gene Ontology GO:0090140 gene signature (https://geneontology.org/). Metacells from the analyzed datasets were scored using standard Scanpy scoring based on the above signatures.

### Metabolic dysregulation

To evaluate the extent of metabolic dysregulation in cells, we employed mitochondrial-localized metabolic pathways curated in MitoCarta [50], focusing on genes that reside within mitochondria. We calculated pathway scores for metacells using standard Scanpy scoring using the genes involved in the respective MitoCarta pathways and compared median scores between HighMT and LowMT metacells. Each pathway was characterized by the vector representing the difference between the median scores of HighMT and LowMT metacells. Hierarchical clustering was performed on these pathway vectors across different cancer types using Ward linkage based on Euclidean distances.

Furthermore, to assess the activation of xenobiotic metabolism, we examined genes involved in three phases of this process: phase I enzymes, predominantly cytochrome P450 enzymes involved in oxidation; phase II enzymes, which conjugate phase I metabolites with molecules like glutathione and sulfate to produce hydrophilic compounds; and phase III proteins, primarily ABC transporters facilitating the transport of drugs across cellular membranes [59]. We compared the expression levels of these genes between HighMT and LowMT metacells across all included studies.

### Link between pctMT and drug resistance in cell lines

To investigate the association between higher pctMT and drug resistance or sensitivity, we used paired RNA-seq and drug sensitivity data from the Cancer Cell Line Encyclopedia (CCLE) [61]. First, we extracted raw RNA-seq counts to calculate pctMT for each cell line. Then, we evaluated the correlation between pctMT and the half-maximal inhibitory concentration (IC50) values of all drugs across the dataset for each cancer type. The median correlation across cell lines within each cancer type was computed, identifying the top 15 drugs with the highest and lowest median correlations as the most resistant and most sensitive drugs, respectively.

Drugs were categorized based on their target disruptions; we compared the distribution of these categories between the full set of drugs tested in CCLE and the most resistant or sensitive drugs using the Fisher exact test. This analysis allowed us to evaluate whether specific categories of drug targets were disproportionately represented among the identified resistant or sensitive drugs across cancer types.

### Link between pctMT and drug resistance in single-cell lineage tracing data

UMI count data for the Kuramochi [72] treatment-naive, carboplatin-treated (1.2 μM, for 3 days), and olaparib-treated (1.2 μM, for 7 days) cells were downloaded from GSE223003, along with associated metadata assigning cells to treatment-sensitive and resistant groups. Data from two replicates for each treatment was merged into a single dataset, and the distribution of pctMT in treatment-naive and post-treatment cells was compared using Mann–Whitney *U* test.

Similarly, UMI count data for the MDAMB468 treatment-naive (control) cells was downloaded from GSE228382. Annotation of treatment-sensitive and treatment-resistant clones was obtained from Table S3 in [73]. Data from replicates of treatment-naive cells was merged into a single dataset, and the distribution of pctMT across treatment-sensitive and resistant clones was compared using Mann–Whitney *U* test. Afatinib-resistant and sensitive clones were identified in the original study by tracking the barcodes of clones present in culture after 40 days of treatment with increasing doses of afatinib (from 250 to 2000 nM by day 40) back to treatment-naive cells. In our comparison, we distinguished between less frequent clones and the two dominant clones (bc14-013:bc30-092942 and bc14-013:bc30-092942), which comprised 81% of all afatinib-tolerant persistent cells identified at day 40 [73].

### Link between pctMT and previously reported transcriptional cell states

We assess the association between pctMT in malignant cells and expression of cancer type-specific transcriptional states by scoring the expression of respective gene signatures. The signatures were scored using standard Scanpy scoring in metacells, and the difference in score distributions between LowMT and HighMT malignant cells was calculated using the Mann–Whitney *U* test.

### Link between pctMT and clinical information in analyzed single-cell studies

To assess the association between the prevalence of HighMT malignant cells and patient clinical features, we calculated a proportion of HighMT cells within the malignant compartment for each patient and associated it with available clinical features reported in the original studies. The difference between the distributions of the proportion of HighMT cells in each clinical category was evaluated using the Mann–Whitney *U* test.

## Supplementary Information

Additional File 1: Supplementary Tables S1 and S2. The file contains descriptions of the mitochondrial genes included in each dataset used in the study, as well as expression of the oxidative phosphorylation program.

Additional File 2: Supplementary Figures S1-S21. The file contains additional information about analyses conducted in the study across all datasets.

## Data Availability

The single-cell studies used in this study can be downloaded from: • The Gene Expression Omnibus (GEO) website: Breast cancer, Wu et al., at GSE176078 [88] and EGAD00001007495 [89]; Pancreatic ductal adenocarcinoma, Steele et al., at GSE155698 [90]; Prostate cancer, Song et al., at GSE176031 [91]; Nasopharyngeal carcinoma, Chen et al., at GSE150430 [92]; Breast cancer, Chung et al., at GSE75688 [93] • The Broad single-cell portal: Metastatic Pancreatic cancer, Raghavan et al. at https://singlecell.broadinstitute.org/single_cell/study/SCP1644/microenvironment-drives-cell-state-plasticity-and-drug-response-in-pancreatic-cancer [94]; Renal clear cell cancer, Bi et al. at https://singlecell.broadinstitute.org/single_cell/study/SCP1288/tumor-and-immune-reprogramming-during-immunotherapy-in-advanced-renal-cell-carcinoma#study-summary [95] • The Curated Cancer Cell Atlas (3CA): Small cell lung cancer, Chan et al. [24], at https://www.weizmann.ac.il/sites/3CA/lung; Lung adenocarcinoma, Bischoff et al. [27], https://www.weizmann.ac.il/sites/3CA/lung; Uveal Melanoma, Durante et al. [28], https://www.weizmann.ac.il/sites/3CA/othermodels •Zenodo: mtDNA-linked single-cell, Kim et al., https://doi.org/10.5281/zenodo.10498240 [96] •Single-cell lineage tracking data: Kuramochi cell line at GSE223003 [97]; MDAMB468 cell line at GSE228382 [98] The bulk data used in this study can be downloaded from: • The Gene Expression Omnibus (GEO) website: Breast cancer, Wu et al., at GSE176078 [88]; Breast cancer, Chung et al., at GSE75688 [93] •The Cancer Cell Line Encyclopedia (CCLE): for the CCLE RNA-seq and drug sensitivity data https://depmap.org/portal/data_page/?tab=allData [99] The two samples processed with spatial transcriptomics method Visium HD are freely available on the 10X website: DCIS https://www.10xgenomics.com/datasets/visium-hd-cytassist-gene-expression-human-breast-cancer-fresh-frozen [100] and LUAD at https://www.10xgenomics.com/datasets/visium-hd-cytassist-gene-expression-human-lung-cancer-post-xenium-expt [101]

## Code availability

The code used to analyze the data and arrive at the conclusions of the study is deposited at Zenodo under the MIT license (DOI: 10.5281/zenodo.15044393) [102] and at https://github.com/BoevaLab/MTRNA-sc-cancer [103].
