# Supplementary material for: Filtering cells with high mitochondrial content depletes viable metabolically altered malignant cell populations in cancer single-cell studies
Source: Genome Biol. 2025 Apr 9;26:91. doi: 10.1186/s13059-025-03559-w (PMC11983838; doi:10.1186/s13059-025-03559-w)
Supplement: Supplementary file 2 — Additional File 2: Supplementary Figures S1-S21. The file contains additional information about analyses conducted in the study across all datasets. [file 13059_2025_3559_MOESM2_ESM.pdf]

Additional file 1: Supplementary Figures

Filtering cells with high mitochondrial content depletes viable metabolically altered malignant cell populations in cancer single-cell studies

Josephine Yates*, Agnieszka Kraft*, Valentina Boeva

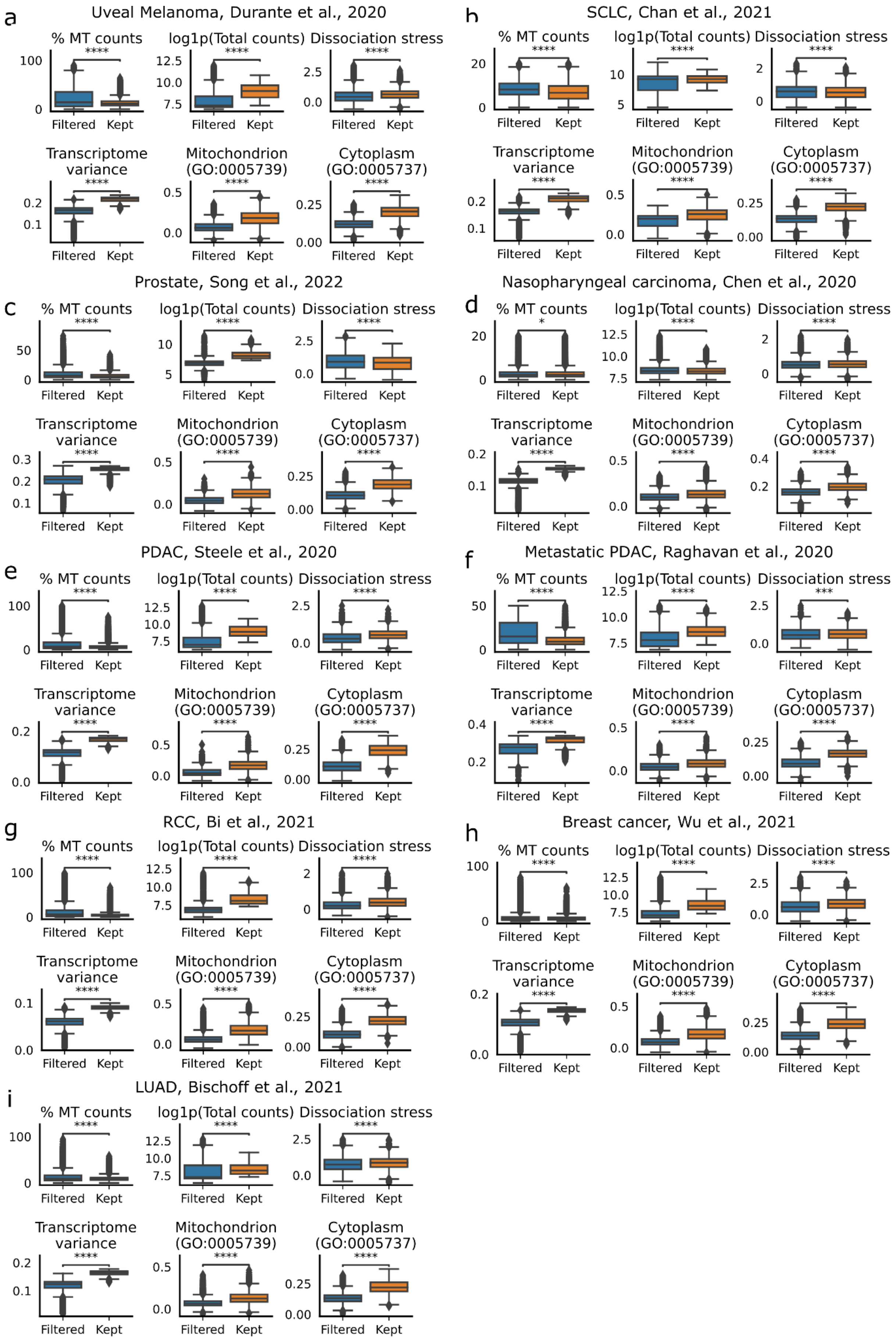
a
Uveal Melanoma, Durante et al., 2020
% MT counts
log1p(Total counts)
Dissociation stress
Transcriptome variance
Mitochondrion (GO:0005739)
Cytoplasm (GO:0005737)
Filtered
Kept
h
SCLC, Chan et al., 2021
c
Prostate, Song et al., 2022
d
Nasopharyngeal carcinoma, Chen et al., 2020
e
PDAC, Steele et al., 2020
f
Metastatic PDAC, Raghavan et al., 2020
g
RCC, Bi et al., 2021
h
Breast cancer, Wu et al., 2021
i
LUAD, Bischoff et al., 2021

Supplementary Figure S1: Distribution of quality metrics of filtered and kept cells using our in-house procedure. **a-h,** For each study included in the analysis [1–9], we run the QC procedure described in Methods, that uses exhaustive QC that does not include using the percentage of mitochondrial counts (% MT counts). We visualize quality metrics for the cells removed with this procedure (Filtered) and those used for the rest of the analysis (Kept). Metrics include standard QC metrics (%MT counts, log1p(total counts)), dissociation stress (computed with the meta-signature devised using the three dissociation stress signatures [10–12]), and three metrics described in Ilicic et al. as cell-type agnostic measures to remove broken and empty droplets (transcriptome variance, mitochondria-located proteins Gene Ontology GO:0005739 and cytoplasm-located proteins GO:0005737). Significance is tested using a Mann-Whitney U test. *: $0.01 \leq p < 0.05$; *:* $0.0001 \leq p < 0.001$***;*** ****: $p < 0.0001$.

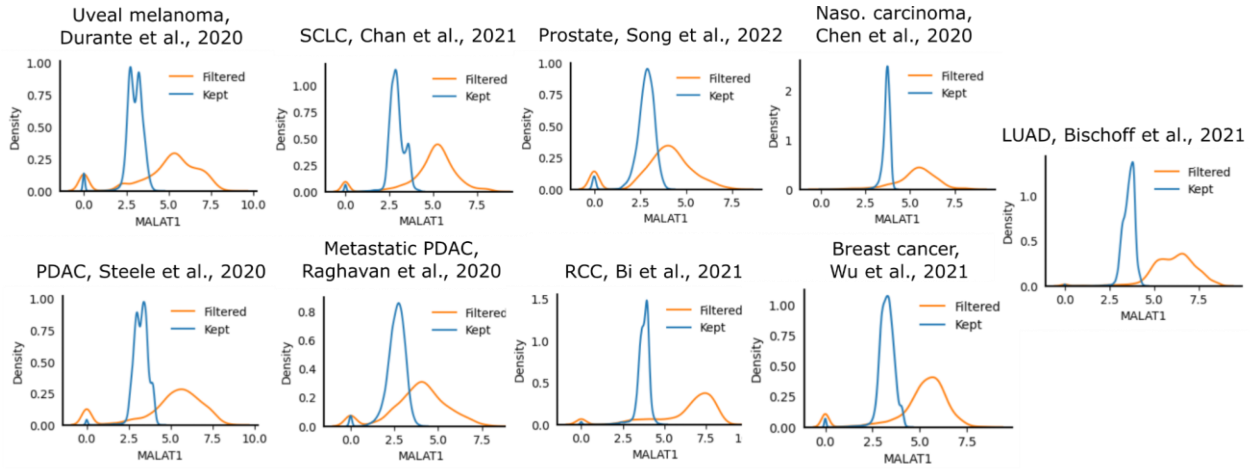

Supplementary Figure S2: MALAT1 expression distribution in filtered and kept cells. For each study included in the analysis, we compare the distribution of MALAT1 expression in Filtered and Kept cells.

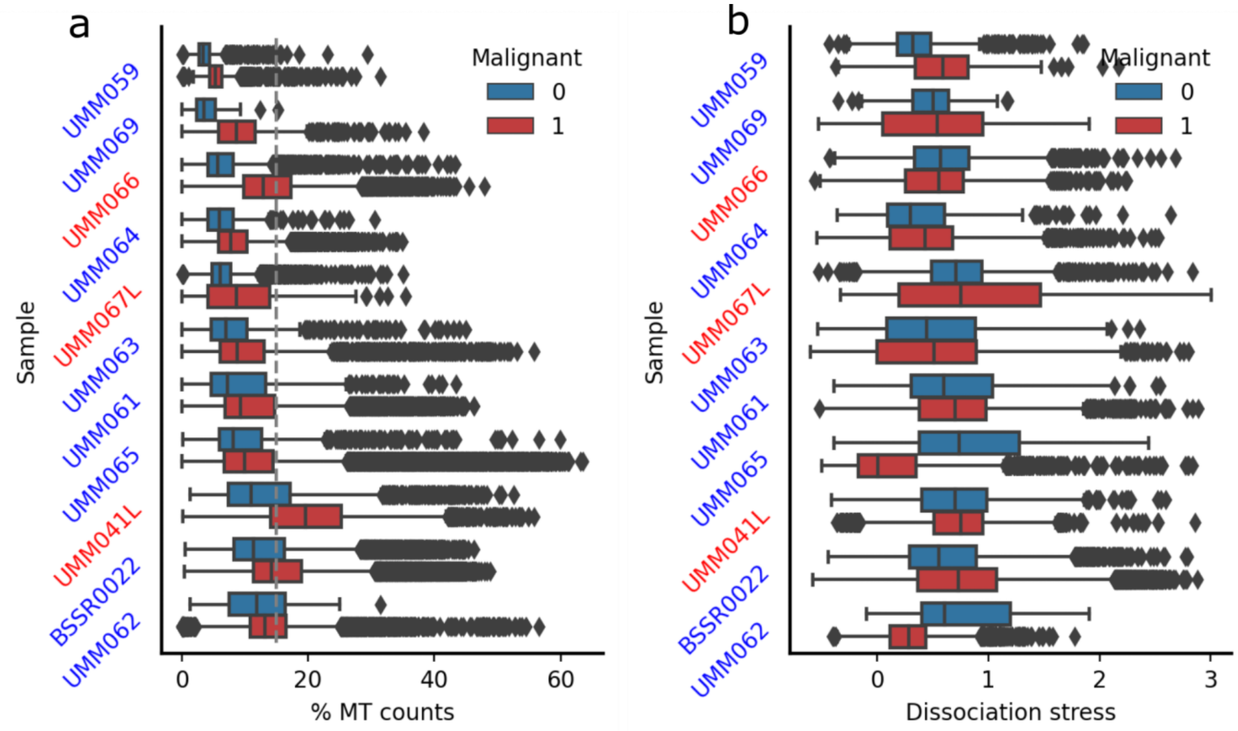

Supplementary Figure S3: Distribution per patient of the uveal melanoma study [8]**, with a, percentage of mitochondrial counts among malignant and TME cells and b, dissociation stress among malignant and TME cells**. Dissociation stress is measured by the score of the meta-dissociation stress signature devised as the genes commonly found in all signatures of dissociation (Methods).

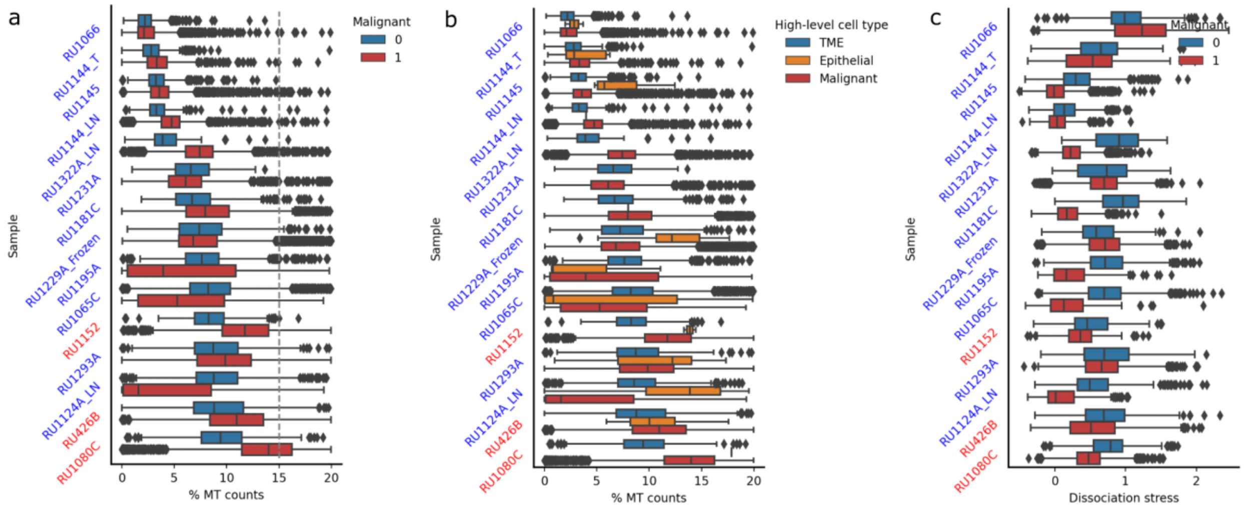

Supplementary Figure S4: Distribution per patient of the small cell lung cancer Chan *et al.* study [1]**, with a, percentage of mitochondrial counts among malignant and TME cells, b, percentage of mitochondrial counts with the separation of TME and normal epithelial cells, and c, dissociation stress among malignant and TME cells**. Dissociation stress is measured by the score of the meta-dissociation stress signature devised as the genes commonly found in all signatures of dissociation (Methods).

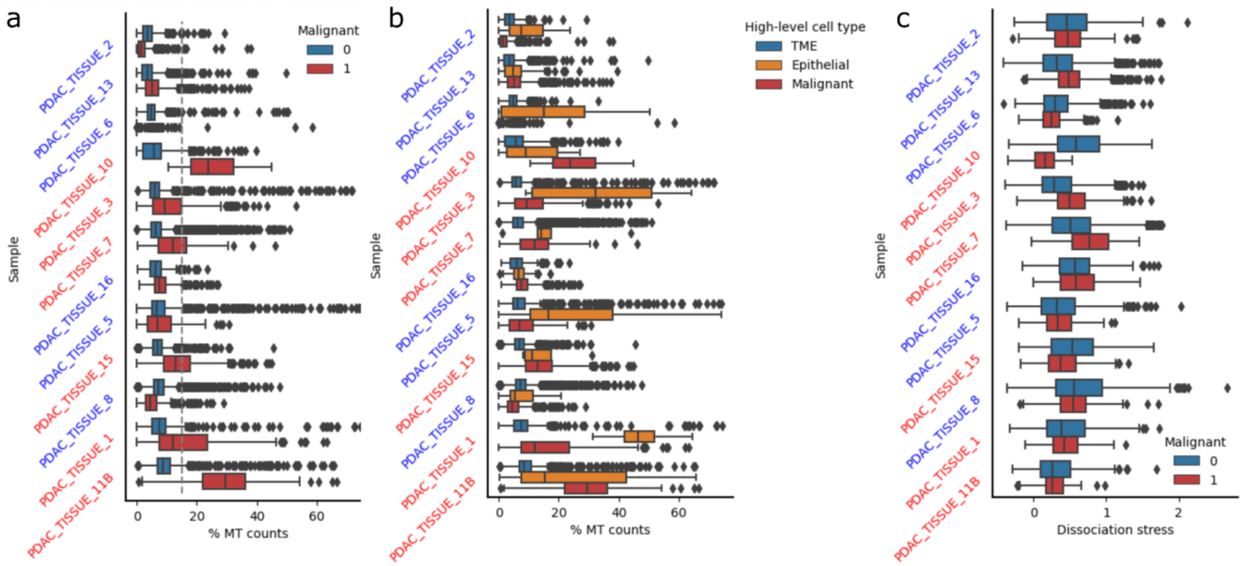

Supplementary Figure S5: Distribution per patient of the pancreas ductal adenocarcinoma Steele *et al.* study [4]**, with a, percentage of mitochondrial counts among malignant and TME cells, b, percentage of mitochondrial counts with the separation of TME and normal epithelial cells, and c, dissociation stress among malignant and TME cells**. Dissociation stress is measured by the score of the meta-dissociation stress signature devised as the genes commonly found in all signatures of dissociation (Methods).

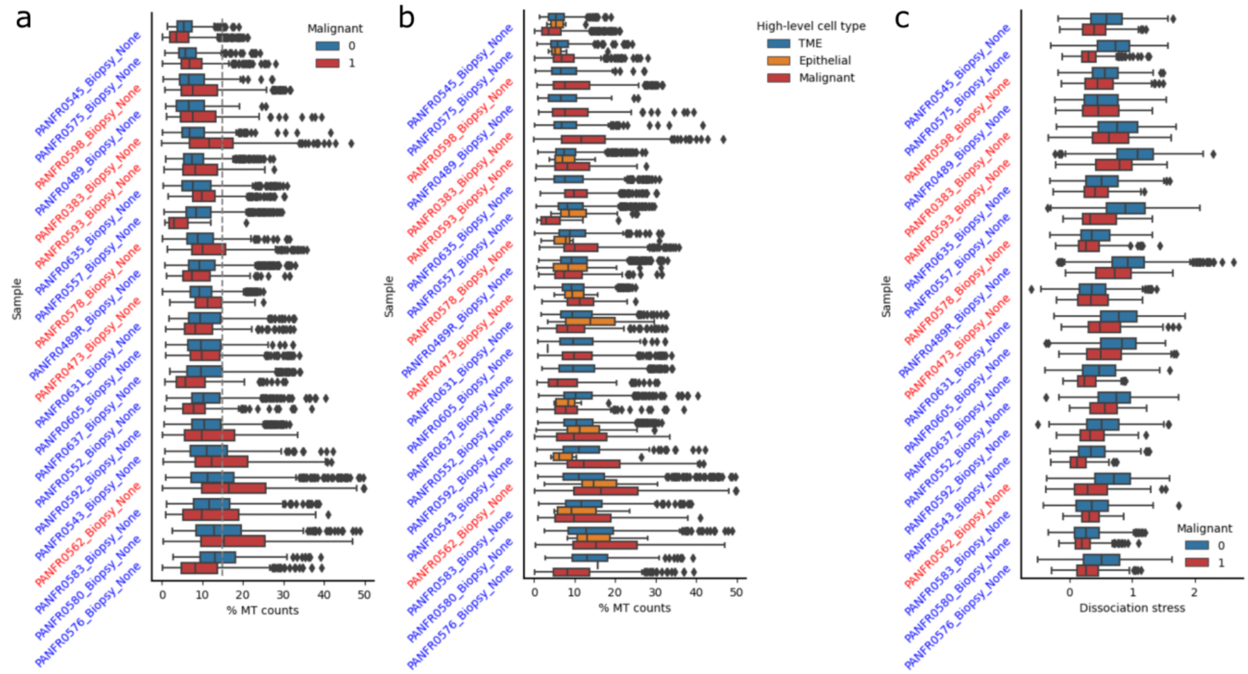

Supplementary Figure S6: Distribution per patient of the metastatic pancreas ductal adenocarcinoma Raghavan *et al.* study [5]**, with a, percentage of mitochondrial counts among malignant and TME cells, b, percentage of mitochondrial counts with the separation of TME and normal epithelial cells, and c, dissociation stress among malignant and TME cells**. Dissociation stress is measured by the score of the meta-dissociation stress signature devised as the genes commonly found in all signatures of dissociation (Methods).

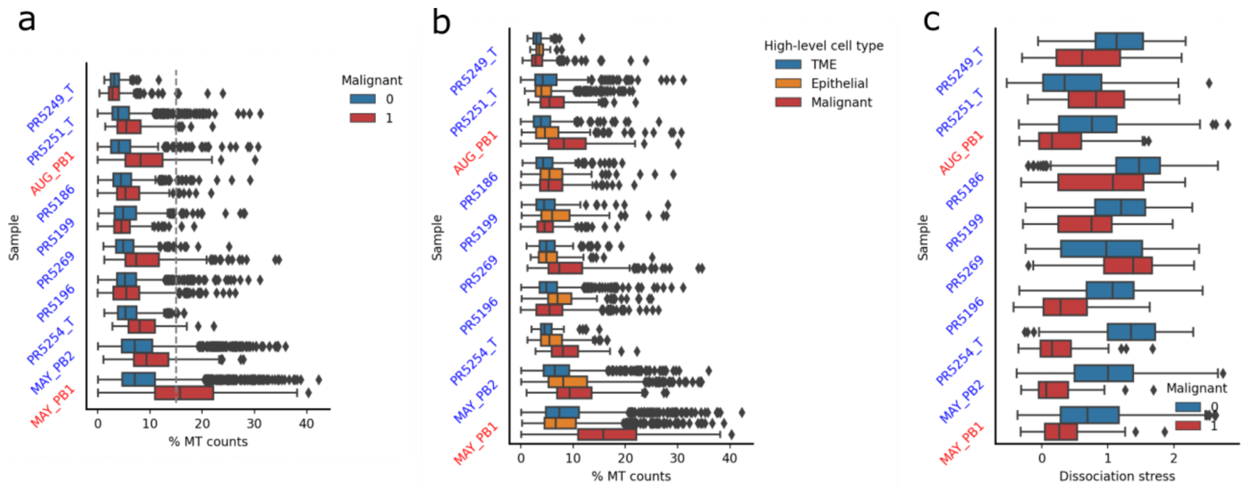

Supplementary Figure S7: Distribution per patient of the prostate cancer Song *et al.* study [2]**, with a, percentage of mitochondrial counts among malignant and TME cells, b, percentage of mitochondrial counts with the separation of TME and normal epithelial cells, and c, dissociation stress among malignant and TME cells**. Dissociation stress is measured by the score of the meta-dissociation stress signature devised as the genes commonly found in all signatures of dissociation (Methods).

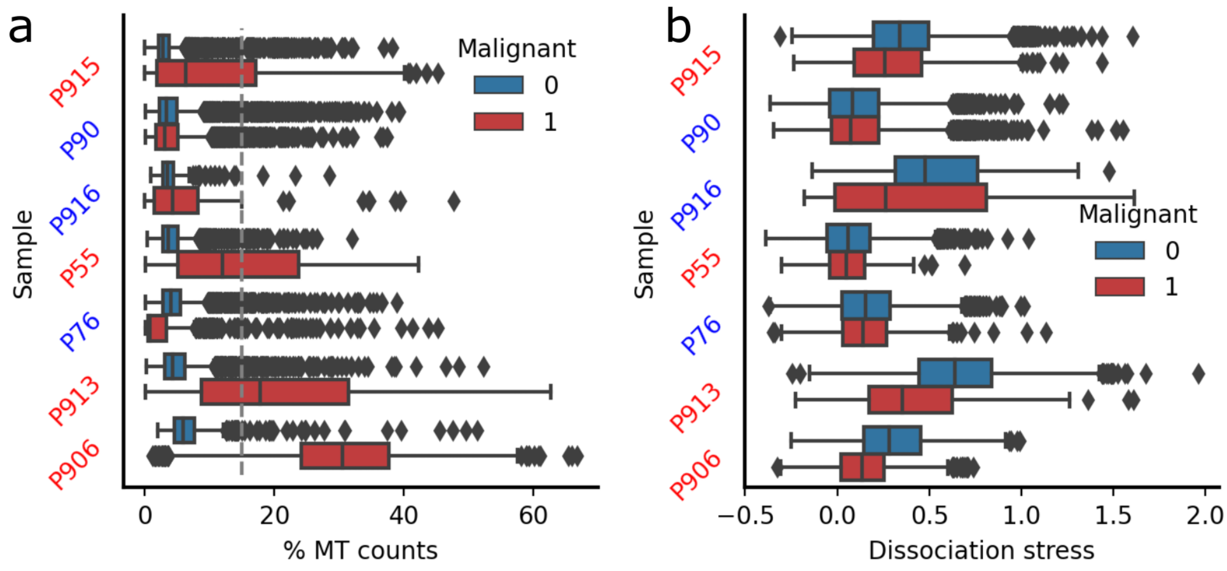

Supplementary Figure S8: Distribution per patient of the renal clear cell cancer Bi *et al.* study [6]**, with a, percentage of mitochondrial counts among malignant and TME cells, and b, dissociation stress among malignant and TME cells**. Dissociation stress is measured by the score of the meta-dissociation stress signature devised as the genes commonly found in all signatures of dissociation (Methods).

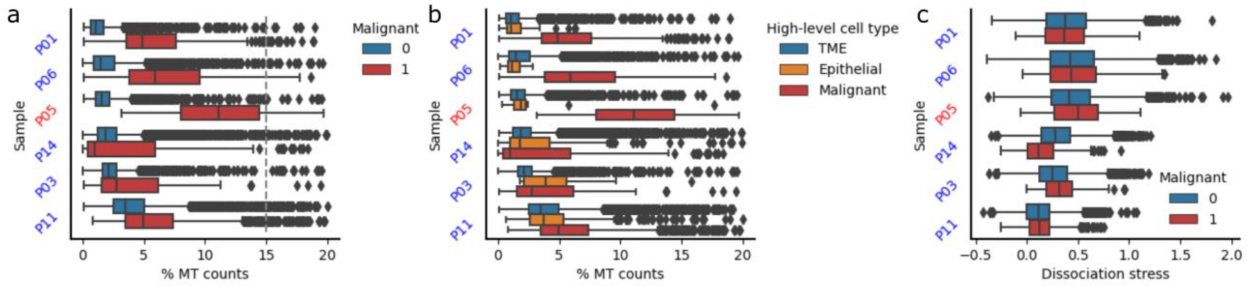

Supplementary Figure S9: Distribution per patient of the nasopharyngeal carcinoma Chen *et al.* study [3]**, with a, percentage of mitochondrial counts among malignant and TME cells, b, percentage of mitochondrial counts with the separation of TME and normal epithelial cells, and c, dissociation stress among malignant and TME cells**. Dissociation stress is measured by the score of the meta-dissociation stress signature devised as the genes commonly found in all signatures of dissociation (Methods).

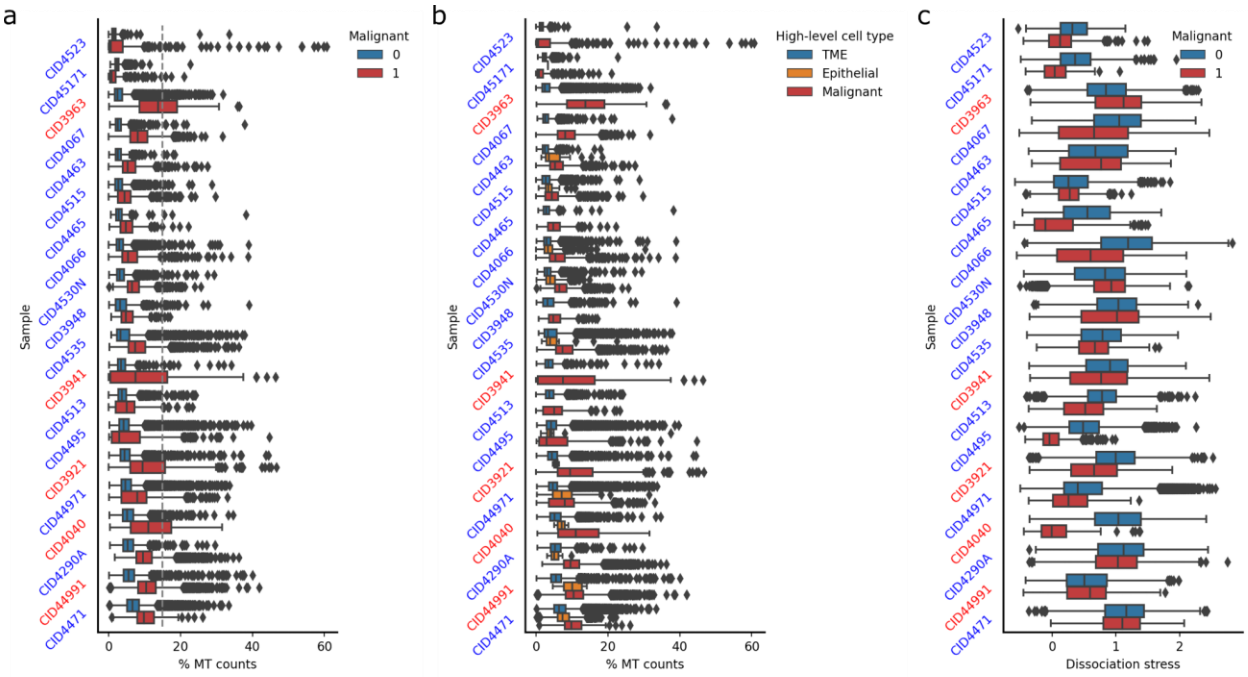

Supplementary Figure S10: Distribution per patient of the breast cancer Wu *et al.* study [7]**, with a, percentage of mitochondrial counts among malignant and TME cells, b, percentage of mitochondrial counts with the separation of TME and normal epithelial cells, and c, dissociation stress among malignant and TME cells**. Dissociation stress is measured by the score of the meta-dissociation stress signature devised as the genes commonly found in all signatures of dissociation (Methods).

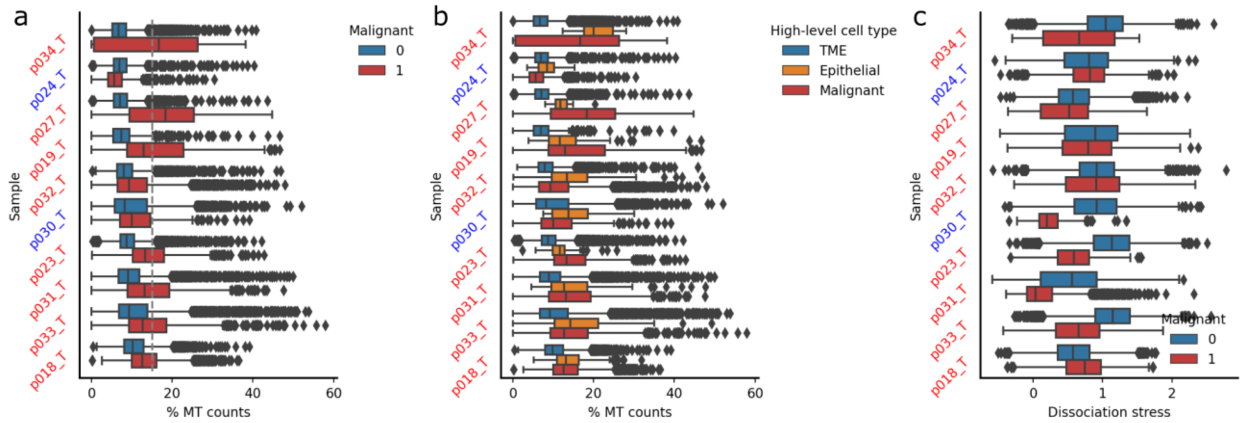

Supplementary Figure S11: Distribution per patient of the lung adenocarcinoma Bischoff *et al.* study [9]**, with a, percentage of mitochondrial counts among malignant and TME cells, b, percentage of mitochondrial counts with the separation of TME and normal epithelial cells, and c, dissociation stress among malignant and TME cells**. Dissociation stress is measured by the score of the meta-dissociation stress signature devised as the genes commonly found in all signatures of dissociation (Methods).

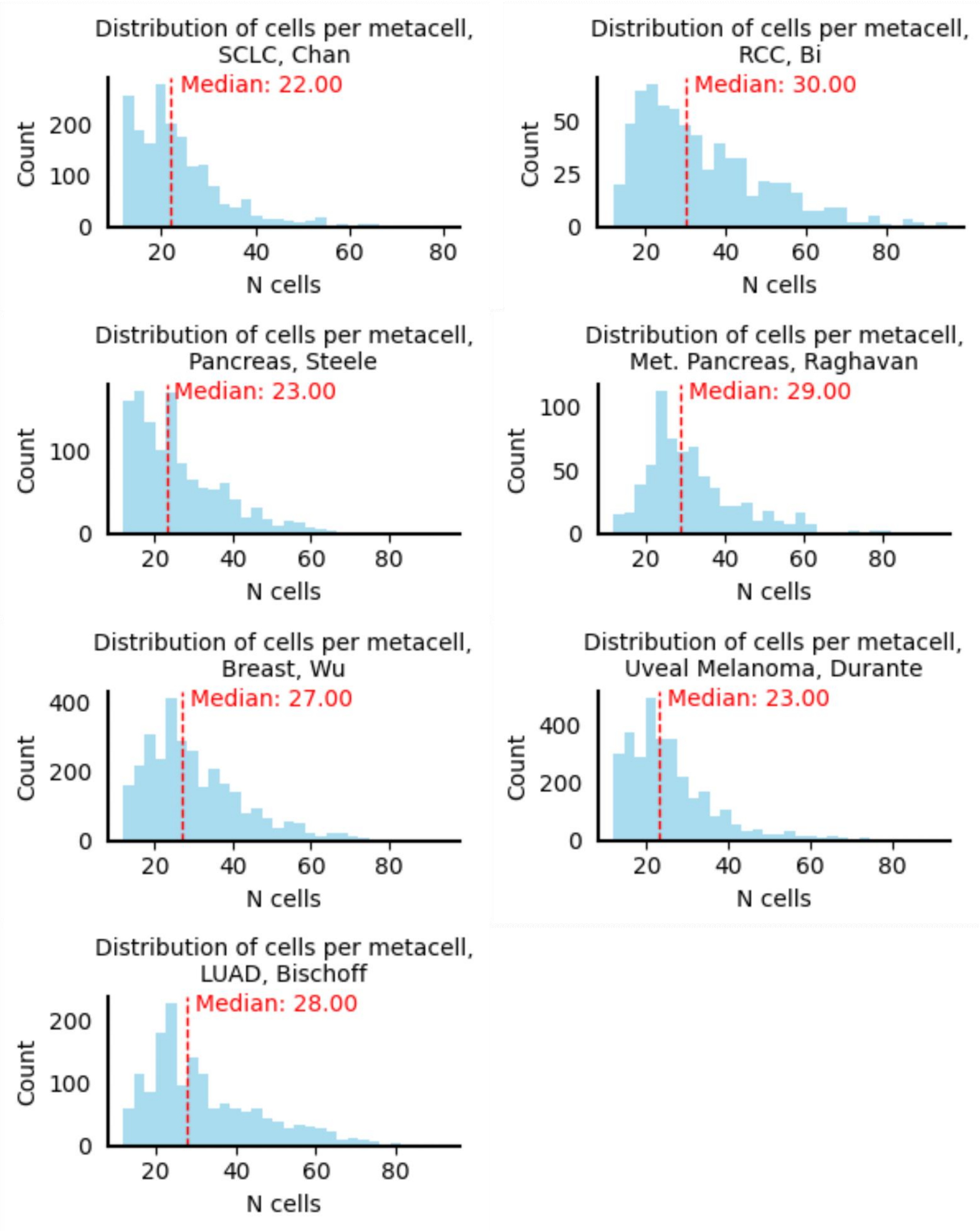

Supplementary Figure S12: Distribution of cell per metacell count across seven studies. Metacells are computed using the metacell package (). The number of cells contained in a metacell are plotted for each study, and the median number of cells per metacell is indicated.

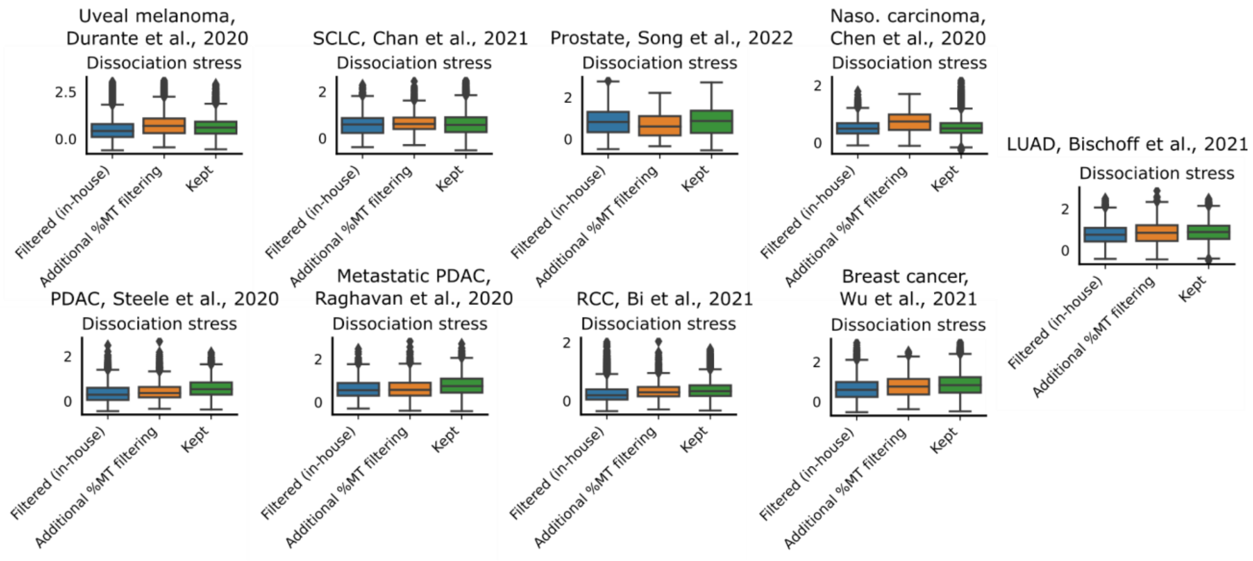

Supplementary Figure S13: Dissociation-induced stress signature expression distribution in filtered and kept malignant cells. For each study included in the analysis, we compute the dissociation stress for cells filtered using our QC procedure, cells kept using our procedure that present >15% MT counts, and all other remaining cells.

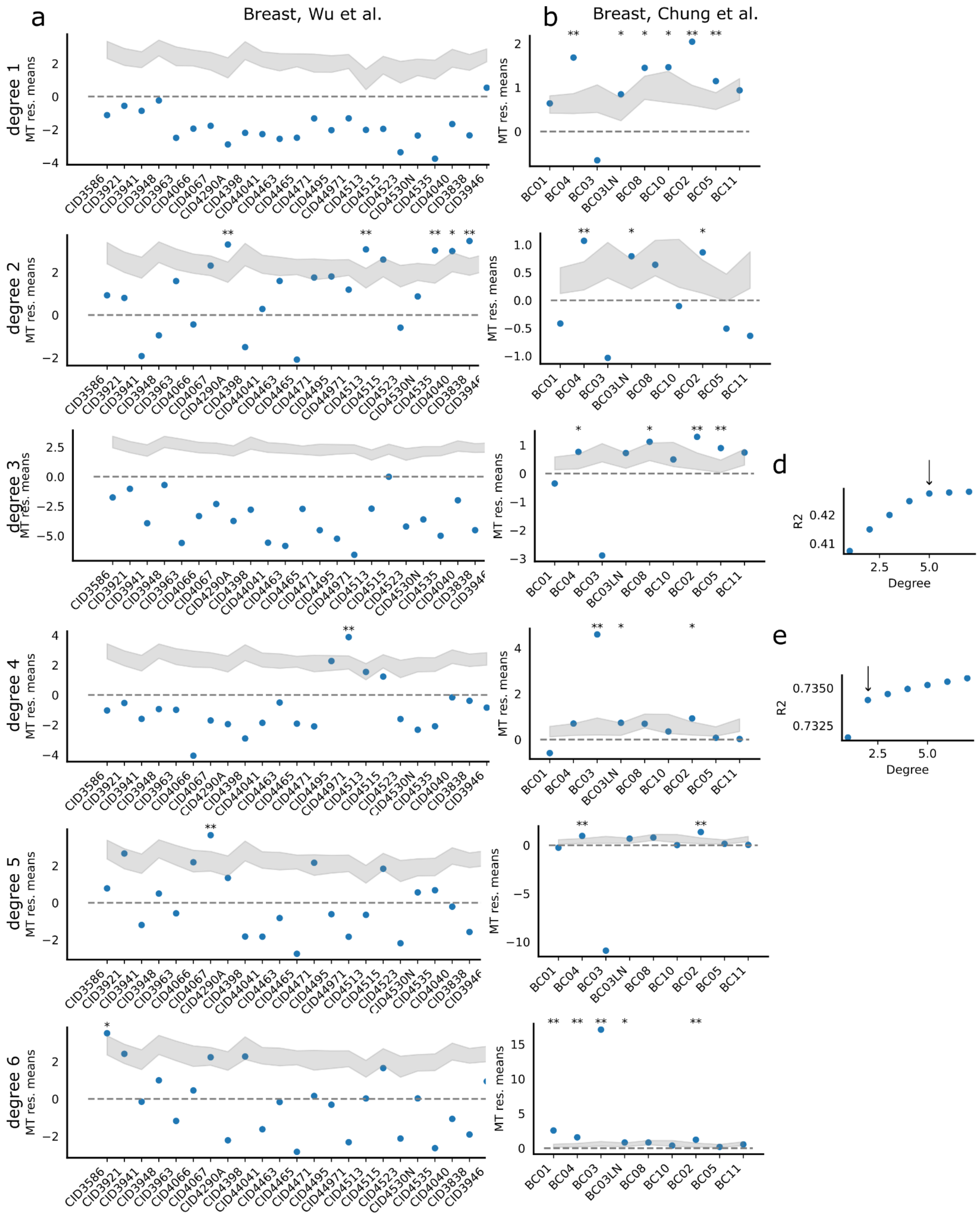

Supplementary Figure S14: Residuals of MT-encoded genes for two breast cancer cohorts across different modelizations for a, the Wu et al. [7] **and b, the Chung et al.** [13] **cohorts.** For each patient, paired bulk and single-cell measurements are available. We model the bulkified vs bulk relationship using a polynomial regression; we represent results for

polynomial regression of degrees 1 through 6. The residuals represent the difference for each MT-encoded gene between the true and predicted bulkified values (Methods). To estimate whether a patient displays significantly differing residuals, we use an empirical one-sided test that tests the amount of times the mean residuals of the MT-encoded genes is higher than that of the randomly sampled genes with a similar level of expression. The 95% confidence interval of the mean residuals of randomly sampled genes is represented as a shaded gray area. **d-e,** Coefficient of determination (R2) for the different degrees for the **d,** Wu et *al.* and **e,** Chung et *al.* cohorts. The selected model is the one at the “elbow”, i.e., when the increased complexity comes at the cost of minimal improvement of R2.

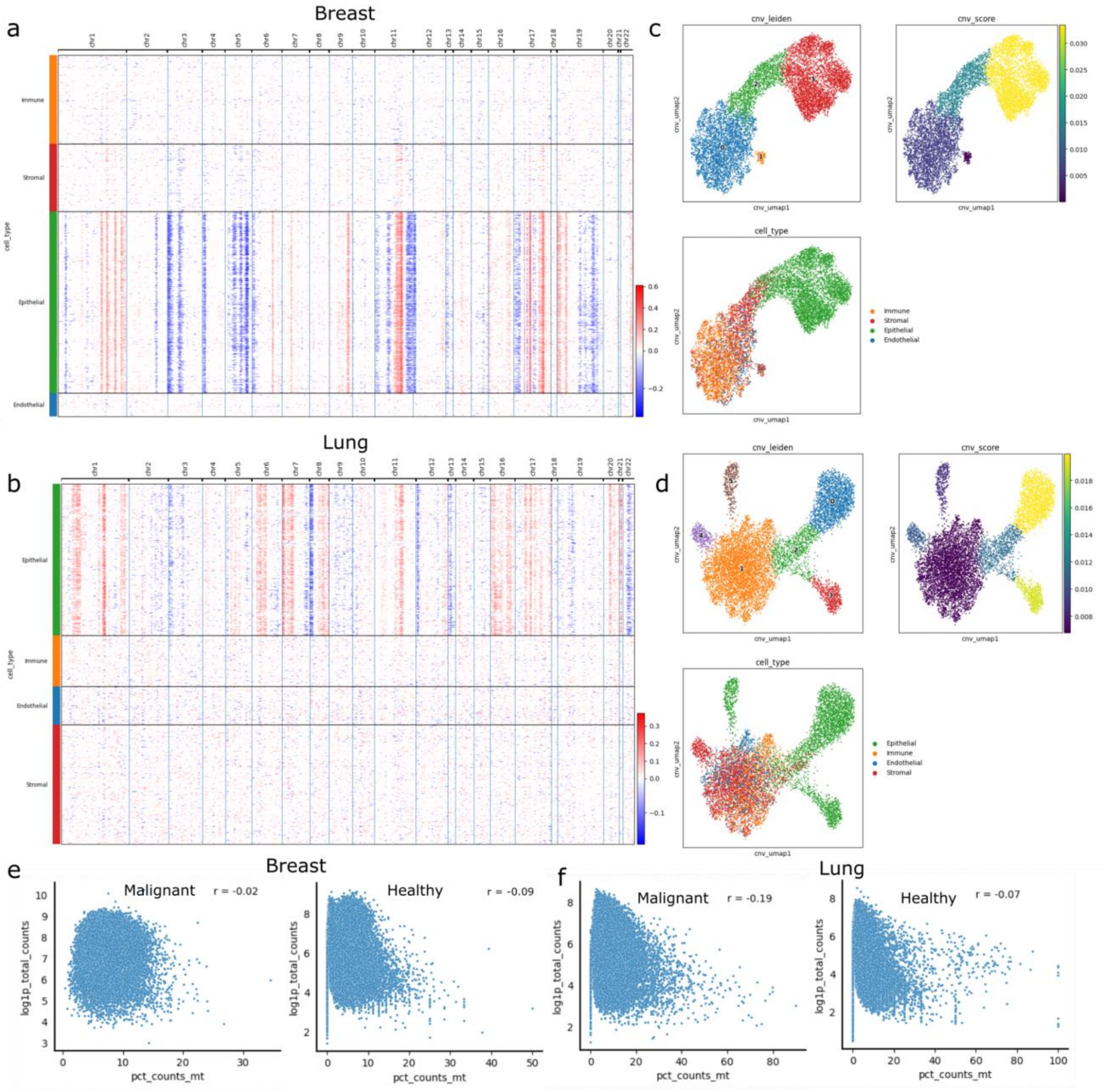

Supplementary Figure S15: **Copy number variation (CNV) analysis of metacells in breast ductal carcinoma in situ (DCIS) and lung adenocarcinoma (LUAD) and correlation between pctMT and total counts. a-b,** CNV profiles of metacells in **a,** DCIS and **b,** LUAD, with cell types assigned based on maximum canonical marker scores and inferCNV analysis using putative healthy cells as a reference. **c-d,** UMAP visualization of metacells with CNV data in **c,** DCIS and **d,** LUAD, colored by Leiden clustering, average CNV score, and putative cell type annotations. CNV clusters are categorized as malignant or healthy based on CNV scores, and cell type annotations are then adjusted accordingly. **e-f,** Correlation between pctMT and log1p of total counts detected in the cell, in malignant and healthy cells, for **e,** DCIS and **b,** LUAD. Pearson's r is indicated on the right.

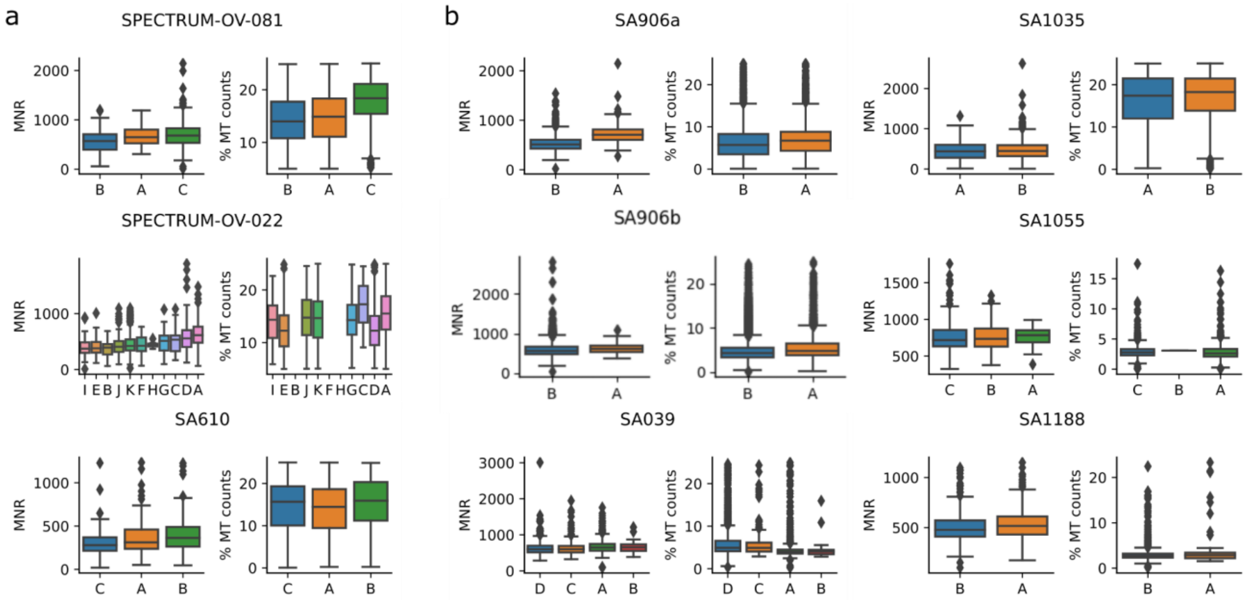

Supplementary Figure S16: Comparison of distribution between mitochondrial-to-nuclear gene ratio (MNR) and the percentage of mitochondrial counts (% MT counts) in **a,** ovarian cancer patient sample and **b,** engineered 184-hTERT cell lines from Kim et al. [14] Cells in scRNA-seq are computationally assigned to clones inferred in the DLP+-sequenced population using the TreeAlign algorithm [15]. The MNR is inferred using the genomic data while the % MT counts is computed using the scRNA-seq data. Clones with higher MNR often exhibit higher % MT counts.

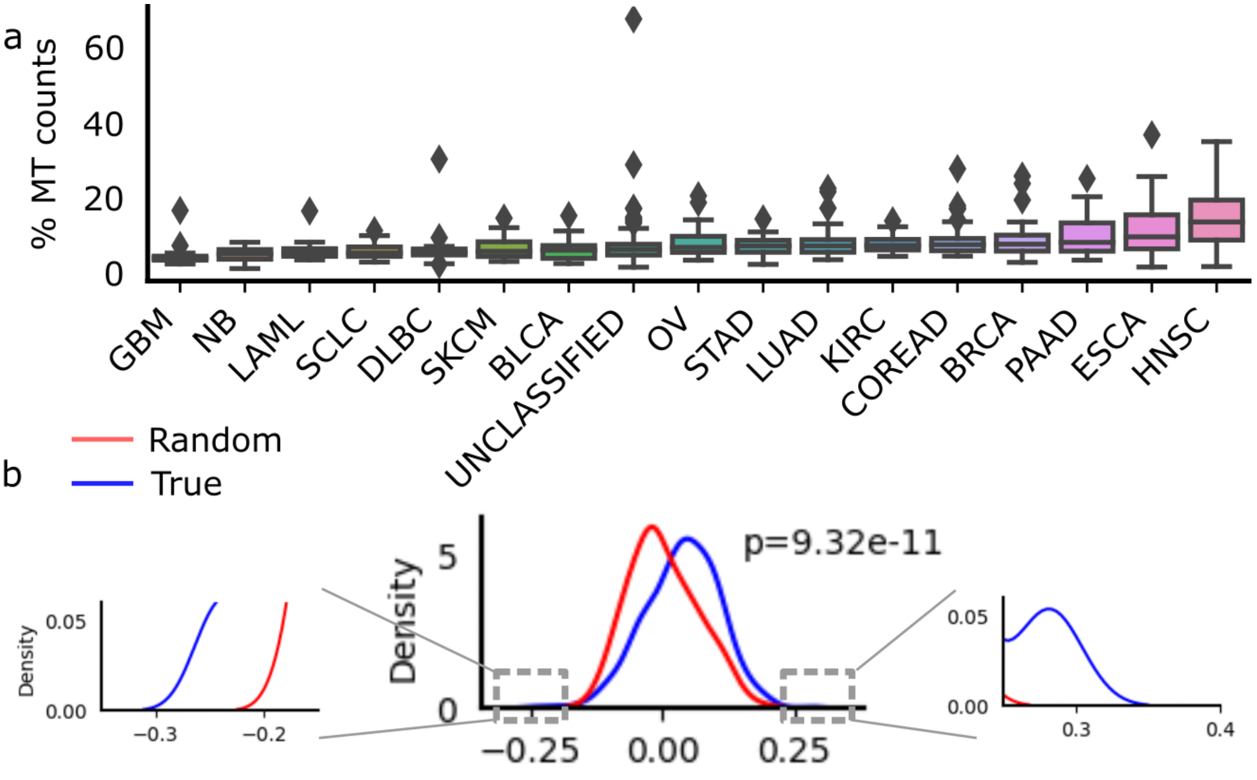

Supplementary Figure S17: Drug resistance in cell lines. a, Distribution of the % of mitochondrial counts in cell lines in the CCLE. **b,** Distribution of the median correlation across cell lines of specific cancer types between the pctMT and the IC50 of drugs. Significance of the difference between distributions is computed using a Kolgomorov-Smirnov test.

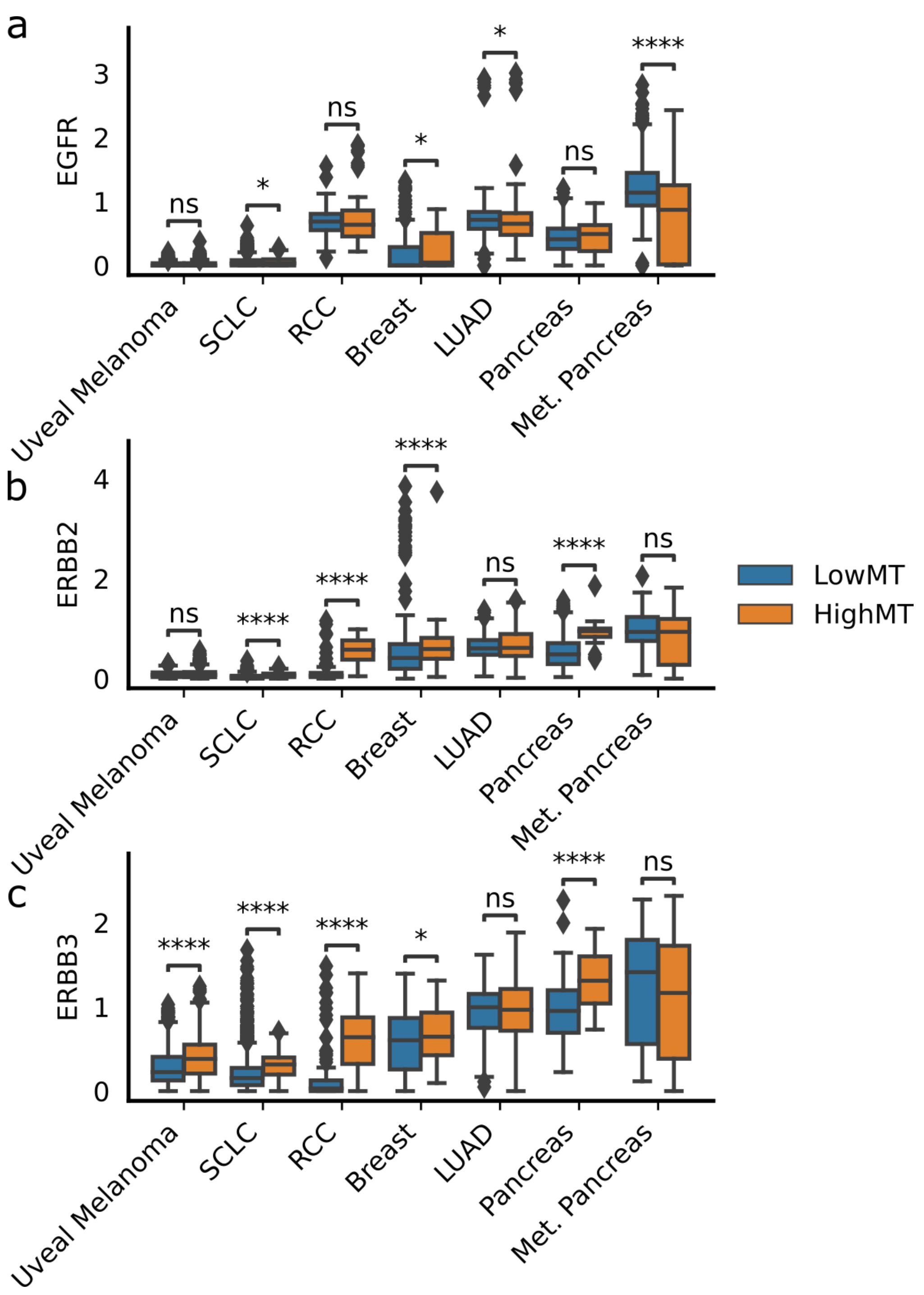

Supplementary Figure S18: EGFR family gene expression across cancer types. The log1p(CP10k) expression distribution is compared across HighMT and LowMT groups using a Mann-Whitney U test.

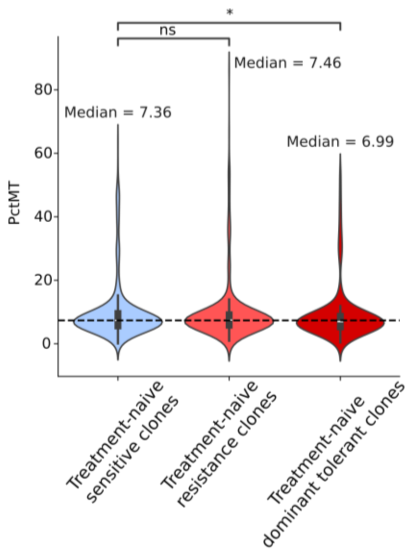

Supplementary Figure S19: Distribution of pctMT across triple-negative breast cancer MDAMB468 cell line's treatment sensitive and resistant clones, treated with Afatinib. Dotted line represents median pctMT of the treatment-naive sensitive clones. The two groups of resistant clones correspond to the ATPC clones (Afatinib Tolerant Persistent Cells) obtained after Day 40 of treatment, with the dominant tolerant clones being the two most frequent resistant clones in the culture after 40 days. Significance is computed using a two-sided Mann-Whitney U test. ns: $p > 0.05$; *: $0.01 \leq p < 0.05$; **: $0.001 \leq p < 0.01$; ***: $p < 0.001$

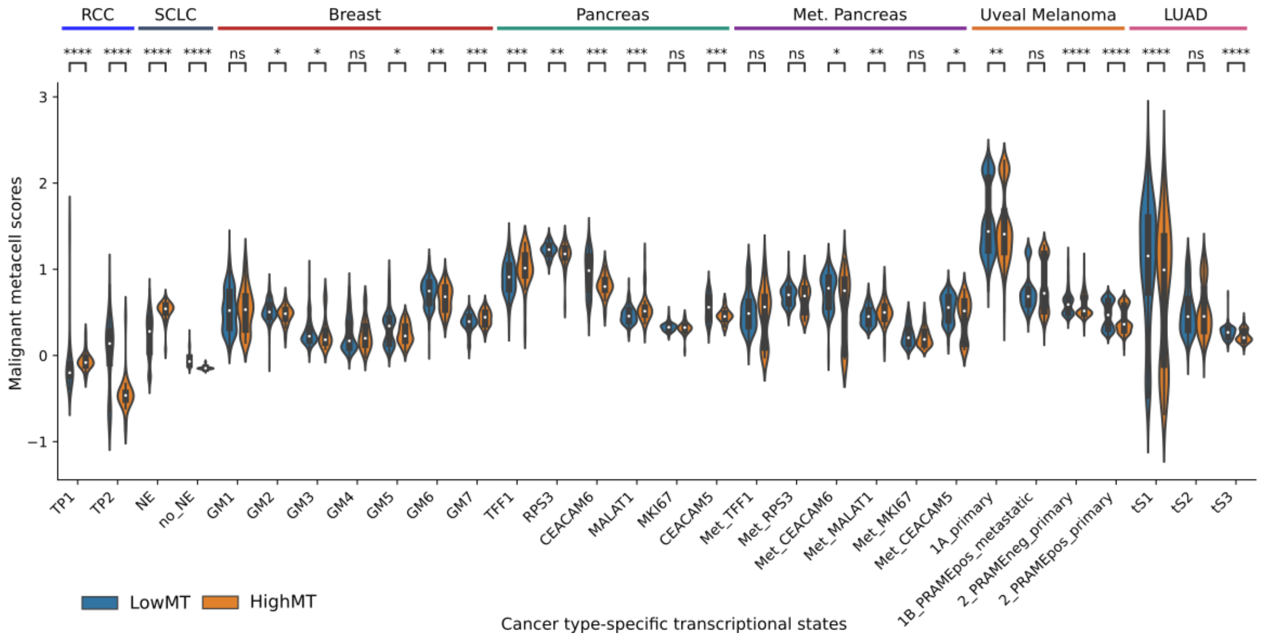

Supplementary Figure S20: Association between pctMT and malignant cell states. a, Distribution of scores of previously reported cancer-specific transcriptional states in LowMT and HighMT malignant cells. Significance is computed using Mann-Whitney U test on metacells. *: $0.01 \leq p < 0.05$; **: $0.001 \leq p < 0.01$; ***: $p < 0.001$.

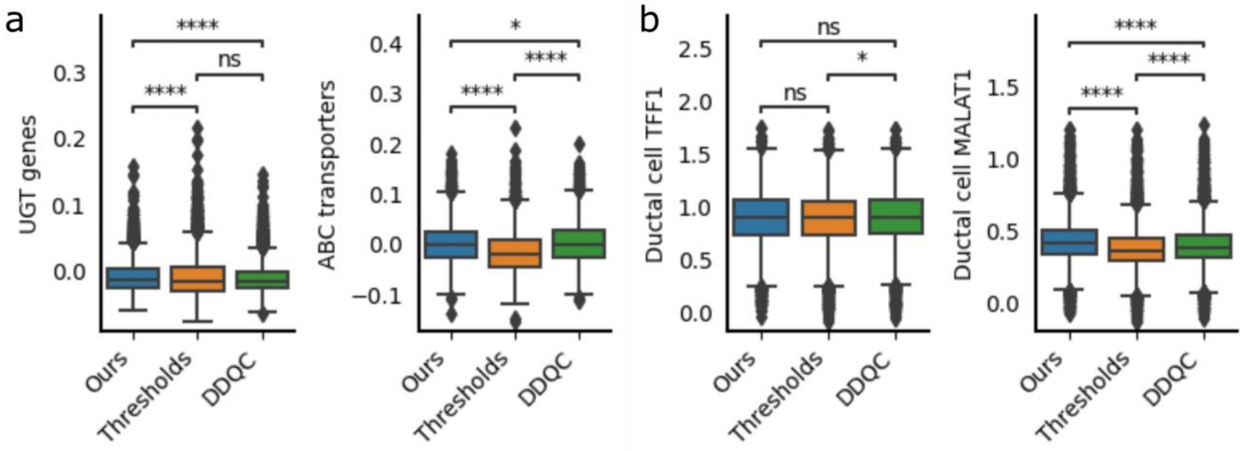

Supplementary Figure S21: Impact of different filtering strategies on downstream analyses. We compare our filtering strategy (Ours), traditional filtering with pctMT thresholding (Thresholds), and a data-driven quality control strategy (DDQC) [16], using the pancreatic cancer Steele *et al.* dataset [4]. **a,** Comparison of UGT and ABC transporters gene expression in the malignant compartment of the pancreatic cancer data, using three different filtering strategies. **b,** Comparison of the signature scores for TFF1 and MALAT1 states described in [17] expression in the malignant compartment of the pancreatic cancer data, using three different filtering strategies. Significance is computed using Mann-Whitney U test. ns: $p > 0.05$; *: $0.01 \leq p < 0.05$; ***: $0.001 \leq p < 0.001$; ****: $p < 0.0001$

References

1. Chan JM, Quintanal-Villalonga Á, Gao VR, Xie Y, Allaj V, Chaudhary O, et al. Signatures of plasticity, metastasis, and immunosuppression in an atlas of human small cell lung cancer. Cancer Cell. 2021;39:1479–96.e18.

2. Song H, Weinstein HNW, Allegakoen P, Wadsworth MH 2nd, Xie J, Yang H, et al. Single-cell analysis of human primary prostate cancer reveals the heterogeneity of tumor-associated epithelial cell states. Nat Commun. 2022;13:141.

3. Chen Y-P, Yin J-H, Li W-F, Li H-J, Chen D-P, Zhang C-J, et al. Single-cell transcriptomics reveals regulators underlying immune cell diversity and immune subtypes associated with prognosis in nasopharyngeal carcinoma. Cell Res. 2020;30:1024–42.

4. Steele NG, Carpenter ES, Kemp SB, Sirihorachai VR, The S, Delrosario L, et al. Multimodal Mapping of the Tumor and Peripheral Blood Immune Landscape in Human Pancreatic Cancer. Nat Cancer. 2020;1:1097–112.

5. Raghavan S, Winter PS, Navia AW, Williams HL, DenAdel A, Lowder KE, et al. Microenvironment drives cell state, plasticity, and drug response in pancreatic cancer. Cell. 2021;184:6119–37.e26.

6. Bi K, He MX, Bakouny Z, Kanodia A, Napolitano S, Wu J, et al. Tumor and immune reprogramming during immunotherapy in advanced renal cell carcinoma. Cancer Cell. 2021;39:649–61.e5.

7. Wu SZ, Al-Eryani G, Roden DL, Junankar S, Harvey K, Andersson A, et al. A single-cell and spatially resolved atlas of human breast cancers. Nat Genet. 2021;53:1334–47.

8. Durante MA, Rodriguez DA, Kurtenbach S, Kuznetsov JN, Sanchez MI, Decatur CL, et al. Single-cell analysis reveals new evolutionary complexity in uveal melanoma. Nat Commun. 2020;11:496.

9. Bischoff P, Trinks A, Obermayer B, Pett JP, Wiederspahn J, Uhlitz F, et al. Single-cell RNA sequencing reveals distinct tumor microenvironmental patterns in lung adenocarcinoma. Oncogene. 2021;40:6748–58.

10. O'Flanagan CH, Campbell KR, Zhang AW, Kabeer F, Lim JLP, Biele J, et al. Dissociation of solid tumor tissues with cold active protease for single-cell RNA-seq minimizes conserved collagenase-associated stress responses. Genome Biol. 2019;20:210.

11. van den Brink SC, Sage F, Vértesy Á, Spanjaard B, Peterson-Maduro J, Baron CS, et al. Single-cell sequencing reveals dissociation-induced gene expression in tissue subpopulations. Nat Methods. 2017;14:935–6.

12. Machado L, Geara P, Camps J, Dos Santos M, Teixeira-Clerc F, Van Herck J, et al. Tissue damage induces a conserved stress response that initiates quiescent muscle stem cell activation. Cell Stem Cell. 2021;28:1125–35.e7.

13. Chung W, Eum HH, Lee H-O, Lee K-M, Lee H-B, Kim K-T, et al. Single-cell RNA-seq enables comprehensive tumour and immune cell profiling in primary breast cancer. Nat Commun. 2017;8:15081.

14. Kim M, Gorelick AN, Vàzquez-García I, Williams MJ, Salehi S, Shi H, et al. Single-cell mtDNA dynamics in tumors is driven by coregulation of nuclear and mitochondrial genomes. Nat Genet. 2024;56:889–99.

15. Shi H, Williams MJ, Satas G, Weiner AC, McPherson A, Shah SP. Allele-specific transcriptional effects of subclonal copy number alterations enable genotype-phenotype mapping in cancer cells. Nat Commun. 2024;15:2482.

16. Subramanian A, Alperovich M, Yang Y, Li B. Biology-inspired data-driven quality control for scientific discovery in single-cell transcriptomics. Genome Biol. 2022;23:267.

17. Zhang S, Fang W, Zhou S, Zhu D, Chen R, Gao X, et al. Single cell transcriptomic analyses implicate an immunosuppressive tumor microenvironment in pancreatic cancer liver metastasis. Nat Commun. 2023;14:5123.
